# Multi-tissue interactions in an integrated three-tissue organ-on-a-chip platform

**DOI:** 10.1038/s41598-017-08879-x

**Published:** 2017-08-18

**Authors:** Aleksander Skardal, Sean V. Murphy, Mahesh Devarasetty, Ivy Mead, Hyun-Wook Kang, Young-Joon Seol, Yu Shrike Zhang, Su-Ryon Shin, Liang Zhao, Julio Aleman, Adam R. Hall, Thomas D. Shupe, Andre Kleensang, Mehmet R. Dokmeci, Sang Jin Lee, John D. Jackson, James J. Yoo, Thomas Hartung, Ali Khademhosseini, Shay Soker, Colin E. Bishop, Anthony Atala

**Affiliations:** 1 Wake Forest Institute for Regenerative Medicine, Wake Forest School of Medicine, Medical Center Boulevard, Winston-Salem, NC 27157 USA; 2 Virginia Tech-Wake Forest School of Biomedical Engineering and Sciences, Wake Forest School of Medicine, Winston-Salem, NC, 27157 USA; 3 Biomaterials Innovation Research Center, Division of Biomedical Engineering, Department of Medicine, Brigham and Women’s Hospital, Harvard Medical School, Cambridge, MA 02139 USA; 4 Harvard-MIT Division of Health Sciences and Technology, Massachusetts Institute of Technology, Cambridge, MA 02139 USA; 5 Wyss Institute for Biologically Inspired Engineering, Harvard University, Cambridge, MA 02139 USA; 6 Center for Alternatives to Animal Testing (CAAT), Bloomberg School of Public Health, Johns Hopkins University Baltimore, 615N Wolfe Street, Baltimore, MD USA; 7 Steinbeis CAAT-Europe, University of Konstanz, Universitätstr 10, Konstanz, Baden-Württemberg Germany; 8 Department of Bioindustrial Technologies, College of Animal Bioscience and Technology, Konkuk University, Seoul, 143-701 Republic of Korea; 9 Department of Physics, King Abdulaziz University, Jeddah, 21569 Saudi Arabia

## Abstract

Many drugs have progressed through preclinical and clinical trials and have been available – for years in some cases – before being recalled by the FDA for unanticipated toxicity in humans. One reason for such poor translation from drug candidate to successful use is a lack of model systems that accurately recapitulate normal tissue function of human organs and their response to drug compounds. Moreover, tissues in the body do not exist in isolation, but reside in a highly integrated and dynamically interactive environment, in which actions in one tissue can affect other downstream tissues. Few engineered model systems, including the growing variety of organoid and organ-on-a-chip platforms, have so far reflected the interactive nature of the human body. To address this challenge, we have developed an assortment of bioengineered tissue organoids and tissue constructs that are integrated in a closed circulatory perfusion system, facilitating inter-organ responses. We describe a three-tissue organ-on-a-chip system, comprised of liver, heart, and lung, and highlight examples of inter-organ responses to drug administration. We observe drug responses that depend on inter-tissue interaction, illustrating the value of multiple tissue integration for *in vitro* study of both the efficacy of and side effects associated with candidate drugs.

## Introduction

The total cost for drug screening and the development process for effective and safe therapeutic agents can exceed 2 billion USD. This includes costs dedicated to target identification, drug screening, regulatory studies, and therapeutic agent manufacturing for clinical trials. Despite extensive preclinical testing and high costs, 90% of drugs that enter Phase I clinical trials ultimately fail^1^. There is a critical need for improved model systems that can accurately predict the effects of drugs, chemicals, and biological agents in the human body^2, 3^.

Traditional *in vitro* two dimensional (2D) cell cultures, currently the standard for early drug compound screening, fail to recapitulate the microenvironment of *in vivo* tissues^4, 5^. Conventional 2D tissue cultures have three major differences from native tissue microenvironments: substrate topography, substrate stiffness, and most importantly, a 2D rather than three dimensional (3D) architecture. As a consequence, 2D culture places a selective pressure on cells, substantially altering their original phenotypic properties. Drug diffusion kinetics are also not accurately modeled in 2D tissue culture, and drug doses effective in 2D are often ineffective when scaled to patients^4, 6, 7^. Animal models serve as the gold standard for biological testing, but have several drawbacks, including uncertainties in interpretation that are rooted in the fact that responses to stimuli in animals are not necessarily predictive of those in humans. Discrepancies in drug activity between human and animal tissues largely result from interspecies differences in metabolism and immunology, contributing to drug attrition rates^8^.

Advancements in cell aggregate culture systems, microengineering, and microfluidic technologies have contributed to the evolution of 3D human tissue-on-a chip models^9, 10^. Currently, many organ-on-a-chip systems are under development^11–17^, as well as a variety of on-chip disease models^11, 18–20^. Despite advances to date, organ-on-a-chip models still lack many of the elements of normal human tissue. In order to improve on currently available technologies, we investigated the use of bioengineered tissues to create a multi-tissue organ-on-a-chip platform. We have previously reported on bioengineered tissues that have been successfully implanted in patients^21–23^, but implementation of highly specialized primary cells like hepatocytes^24^ and cardiomyocytes^25, 26^ for drug discovery applications has proven to be a technically difficult and expensive process^27^. In addition to being able to source adequate tissue-specific human cells for our bioengineered constructs, incorporation of supportive cells (e.g. endothelial cells and fibroblasts) as well as structural matrix proteins can allow for recapitulation of the dynamic interactions of cells with each other and with the cellular microenvironment.

The next challenge is to combine multiple tissue or organ types within a single microfluidic device as a simple model of an organism-on-a-chip for drug and therapeutic studies. This is critical because tissues and organs in the human body are not isolated, but are instead highly interconnected. For example, it is essential that organs receive biochemical signals and support from other organs to function normally. Likewise, in drug screening applications, toxic effects in secondary tissues can be as important as effects at the target site. If undetected, these effects can lead to an unnecessarily high rate of drug failure or withdrawal from the market due to negative side effects. Furthermore, malignant tumor cell metastasis from one tissue to another location is dependent on multiple tissue or organ sites as well as a circulatory system (vascular or lymphatic)^28^. As such, while useful for many applications, single organoid models have limited efficacy for recapitulating the complex physiological interactions between multiple tissues that occur naturally in the human body. Multi-organoid platforms represent a logical progression of organ-on-a-chip technology and will allow for the realization of truly predictive *in vitro* modeling of human physiology.

Currently, organ-on-a-chip systems that comprehensively and accurately mimic human tissues or model diseases are limited, and there are fewer still in which multiple organs are represented in an integrated fashion. In one recent example, a 4-tissue pumpless perfusion system was developed that integrated 2D tissue cultures of liver, cardiac, skeletal muscle, and neuronal components within a single device. This platform was employed to demonstrate basic cell toxicity in drug screens with doxorubicin, atorvastatin, valproic acid, acetaminophen and N-acetyl-m-aminophenol^29^. In another example, a microphysiological fluidic platform was developed containing preformed intestine and skin constructs, liver spheroids, and a kidney epithelial barrier tissue model, in which basic function, gene expression, and viability were maintained for 28 days^30^. These examples represent important steps towards systems that can mimic complex responses and interactions between tissues during drug and toxicology screens, but are also limited in the sense that they utilize 2D cultures or cell lines, rather than higher functioning primary cells or iPS-derived cells, potentially limiting their upper limit of physiologically accurate tissue function.

In this study, we advance integrated technologies further by describing the development and functional integrated testing of more complex bioengineered tissue organoids. Each tissue model was created with the cell types present in the native human tissue, with similar relative proportions where feasible. Bioprinting^31–35^ with customized tissue-specific bioinks^36, 37^ and fluidic device technologies were employed to integrate liver and cardiac tissue organoids precisely and reproducibly into modular perfusable devices that were connected to a lung organoid at the air-liquid interface. This system was then employed to assess physiological responses to drugs and toxic agents. Within this three-tissue organ-on-a-chip system, the three bioengineered tissue organoids are capable of responding to a variety of external stimuli independently or in concert^38^, similar to organ dynamics found in the human body. Advanced biosensors were developed to provide the capability for real time data acquisition. To showcase the utility of this new platform, we demonstrate physiological characterization, functional testing, drug and toxicology screening, and critically, integrated responses to agents that highlight the importance of inter-organ interactions.

## Results

### Overall design of the three-tissue organ-on-a-chip platform

**Figure 1:**
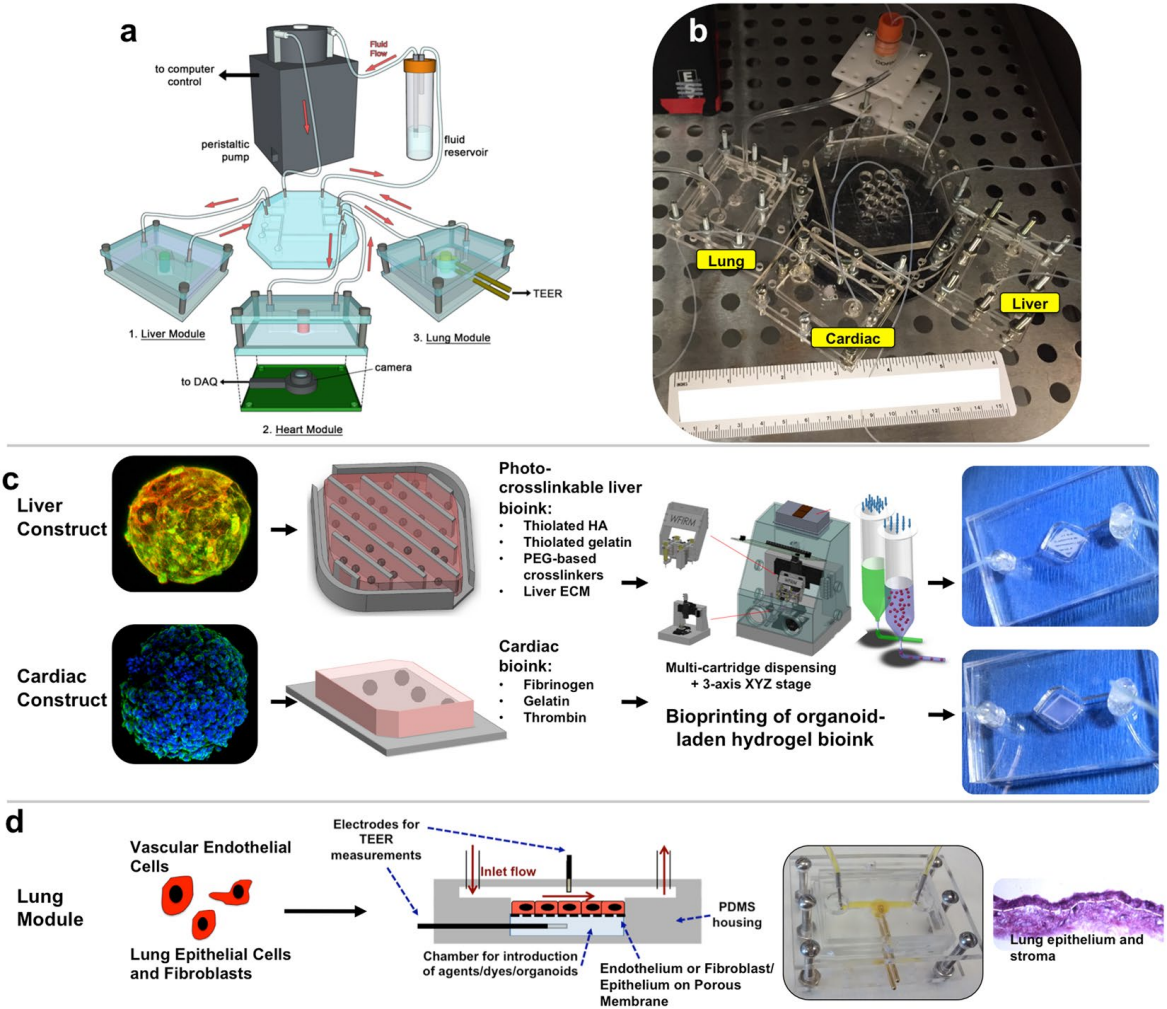
Overall design and implementation strategy for the 3-tissue-representative organ-on-a-chip system using a variety of biofabrication approaches. (**a**,**b**) Illustration and photograph of the modular multi-tissue organ-on-a-chip hardware system set up for maintenance of 3 tissue model. Individual microfluidic microreactor units house each organoid or tissue model, and are connected via a central fluid routing breadboard, allowing for straightforward “plug-and-play” system preparation initialization. (**c**,**d**) General overview of how each tissue type is prepared for the system. (**c**) Liver and cardiac modules are created by bioprinting spherical organoids within customized bioinks, resulting in 3D hydrogel constructs that are placed into the microreactor devices. (**d**) Lung modules are formed by creating layers of cells over porous membranes within microfluidic devices. Introduction of TEER (trans-endothelial [or epithelial] electrical resistance sensors allows monitoring of tissue barrier function integrity over time.

The goal of our work was to develop a highly functional, perfusion-driven, microfluidic multi-tissue organ-on-a-chip system comprised of liver, heart, and lung organoids (Fig. 1a,b). These individual tissue constructs are housed in modular microreactors formed by conventional polydimethyl-siloxane (PDMS) soft lithography and molding^39^. Connected serially under fluid flow (Fig. 1b), this system allows modular “plug and play” capabilities for platform configuration. Moreover, this strategy also supports the integration additional tissues in the future. Because of the specific individual requirements of the different components, a toolbox of biofabrication techniques was employed to create each tissue model (Fig. 1c,d). Spherical liver organoids were formed with primary human hepatocytes, stellate cells, and Kupffer cells, and spherical cardiac organoids were formed using induced pluripotent stem (iPS) cells. These spherical organoids were then bioprinted (Supplementary Fig. 1) into the microreactors using either an ECM-derived bioink^36, 37, 40^ (liver) or a fibrin and gelatin bioink (cardiac), employing hydrogel crosslinking to immobilize the organoids as larger composite constructs within the individual microreactor devices (Fig. 1c). These bioink formulations were previously determined to be most supportive of viability and function of their respective organoid types. Lung modules (Fig. 1d **)** were fabricated in microreactors with a similar form factor, but featuring an immobilized semi-porous membrane on which lung fibroblasts, epithelial, and endothelial cells were seeded in layers. Custom trans-endothelial electrical resistance (TEER) electrodes were integrated into the module for real-time barrier function monitoring. Following biofabrication, devices were sealed and connected to the circulatory perfusion system through a central fluid routing breadboard. Fluid flow was driven by a micro-peristaltic pump. Extensive individual tissue model characterization is described in Supplementary Figs 2–6 and the accompanying Supplementary Results, including histology, immunostaining, metabolic assays, and additional functional tests, demonstrating the physiological relevance of each tissue model type.

### Liver-on-a-chip

**Figure 2:**
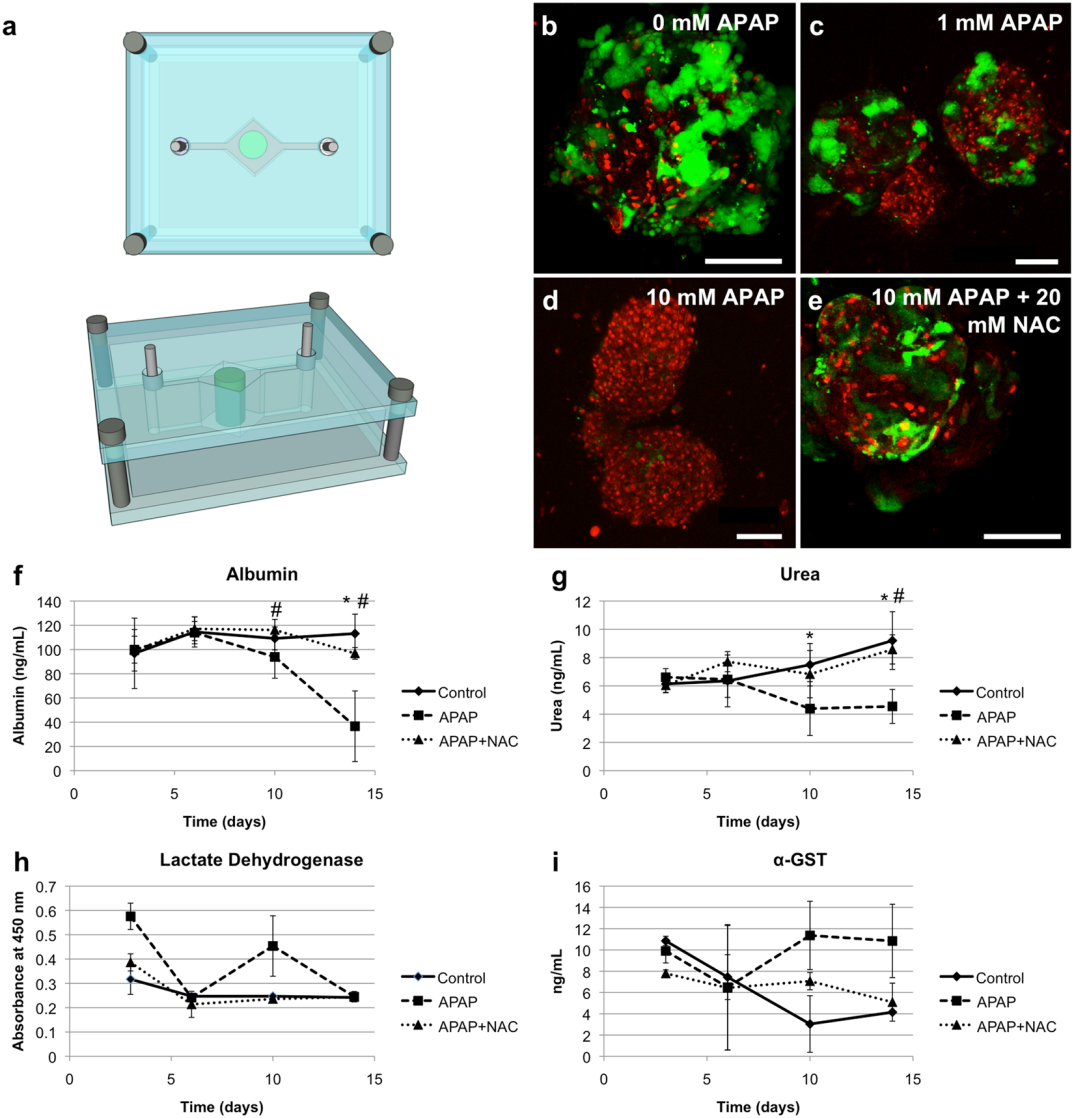
Liver on a chip: On-chip response to acetaminophen, N-acetyl-L-cysteine countermeasure. (**a**) A depiction of the liver-on-a-chip microreactor device. (**b**–**e**) Liver organoids respond to acetaminophen toxicity and are rescued by NAC. Viability as determined by LIVE/DEAD staining on day 14. Organoids were exposed to (**b**) a 0 mM APAP control, (**c**) 1 mM APAP, (**d**) 10 mM APAP, or (**e**) 10 mM APAP with 20 mM N-acetyl-L-cysteine. Green – Calcein AM-stained viable cells; Red – Ethidium homodimer-stained dead cells. (**f**–**i**) Analysis of media aliquots indicate that APAP induces loss of function and cell death, while NAC has the capability to mitigate these negative effects. Only 10 mM APAP treatment data shown. Quantification of (**f**) human albumin, (**g**) urea, (**h**) lactate dehydrogenase, and (**i**) alpha-GST. Albumin and urea output are negatively effected by APAP treatments, while NAC decreases this reduction in secretion. LDH and alph-GST are low in control and APAP + NAC groups demonstrating functional cells, while APAP induces elevated levels, indicating apoptosis and release of LDH and alpha-GST into the media. Statistical significance: *p < 0.05 between control and APAP; ^#^p < 0.05 APAP + NAC and APAP.

The liver-on-a-chip microreactor device (Fig. 2a) was developed first, as liver toxicity and liver metabolism are integral to the cause of many failures in the drug development pipeline^41^. Initial characterization confirmed the stability of organoid size and ATP activity, as well as a variety of markers for hepatocytes, stellate cells, and Kupffer cells (Supplementary Fig. 2a–j). Remarkable viability was confirmed across several time points in culture through 4 weeks (Supplementary Fig. 2k–m). Additionally, functional outputs of urea and albumin were verified, as was the presence of key cytochrome P450 enzymes CYP3A4 and CYP2C19, as demonstrated by the metabolism of diazepam to downstream metabolites temazepam, nordiazepam, and oxazepam (Supplementary Fig. 3). The latter was observed to be exceptionally superior to 2D hepatocyte cultures.

To demonstrate clinical relevance, liver construct toxicity response was assessed following exposure to acetaminophen (APAP) and the clinically-used APAP countermeasure N-acetyl-L-cysteine (NAC). On day 6, liver constructs in the fluidic system were treated with no drug, 1 mM APAP, 10 mM APAP, or 10 mM APAP +20 mM NAC, respectively (n = 6 for each condition). Viability was assessed as described. Based on the ratio of live cells to dead cells. The control group maintained a high level of viability (70–90% at day 14) throughout the 14 day experiment (Fig. 2b). By comparison, the 1 mM APAP group showed reduced viability (30–50%, Fig. 2c) during the same time period, while the 10 mM APAP group had very few viable cells remaining at day 14 (0–1%, Fig. 2d). Addition of NAC maintained the viability associated with 10 mM APAP exposure significantly (50–60%, Fig. 2e), as expected for this APAP countermeasure. Indeed, organoid morphology appeared more like that of constructs that received the lesser 1 mM APAP treatment.

Biochemical assessment of the circulating media for each drug condition was then performed. Albumin analysis revealed constant albumin production by liver organoids through day 6, remaining near 120 ng/mL on average (Fig. 2f). Albumin levels at the first two time points were not statistically significant in comparison to one another, as would be expected at the pre-treatment time points. Following drug administration on day 6, albumin levels were significantly decreased in the 10 mM APAP group compared to the 0 mM control (p < 0.05). Additionally, the 10 mM APAP group albumin levels were significantly decreased compared to the 1 mM APAP group (p < 0.05, *not shown*). At day 14 the albumin levels in the 10 mM APAP group were nearly immeasurable, while levels in the APAP + NAC organoid were significantly greater (p < 0.05) and nearly indistinguishable from control levels. The general trend of the data shows both a significant cytotoxic response to APAP as well as its mitigation by NAC treatment, accurately reflecting the clinical responses seen in human patients. Urea analysis showed similar results (Fig. 2g), yielding insignificant differences among groups during time points prior to APAP administration and dose-dependent reductions following. On day 10 and 14, the urea level for the 0 mM control group and the APAP + NAC organoid did not differ significantly, but were both significantly higher than the 10 mM APAP group (p < 0.05).

Media samples were analyzed for lactate dehydrogenase (LDH) and α-glutathione-S-transferase (α-GST) (Fig. 2h,i), cytoplasmic proteins that indicate liver cell death upon release to the media. There was initial variability in LDH levels on day 3, potentially attributed to stresses placed on the cells during the bioprinting and microfluidic initiation phases. By day 6, all groups were indistinguishable from one another. On day 10, the first collection point after drug administration, the 10 mM APAP group showed a clear increase in LDH concentration in the media, while the APAP + NAC group was almost identical to the control group. The 10 mM APAP group was not significantly different from the other groups on day 10, but the trend was evident. By day 14, the LDH levels drop down to baseline, suggesting the majority of LDH release occurred between day 6 and day 10, resulting in the spike in LDH on day 10 in the APAP group. The organoids in each group secreted similar levels of α-GST at the day 3 and day 6 time points. Detectable levels of α-GST (between 7 and 11 ng/mL) were present on day 3 in all groups and then decreased over time in the control group. Again, this suggests that the bioprinting process and initiation of microfluidic culture may have placed some stress on the cells in the organoids. Following administration of 10 mM APAP, α-GST increased to over 11 ng/mL by day 10, remaining near that level until the end of the experiment. In comparison, the control organoid group showed a decrease in α-GST to less than 4 ng/mL, indicating that APAP did indeed invoke cell death resulting in release of α-GST into the media. Administration of NAC with APAP clearly attenuated the effects of APAP. On day 10 and day 14, α-GST was detected at about 6 and 5 ng/mL, respectively, in APAP + NAC cultures.

#### Heart-on-a-chip

**Figure 3:**
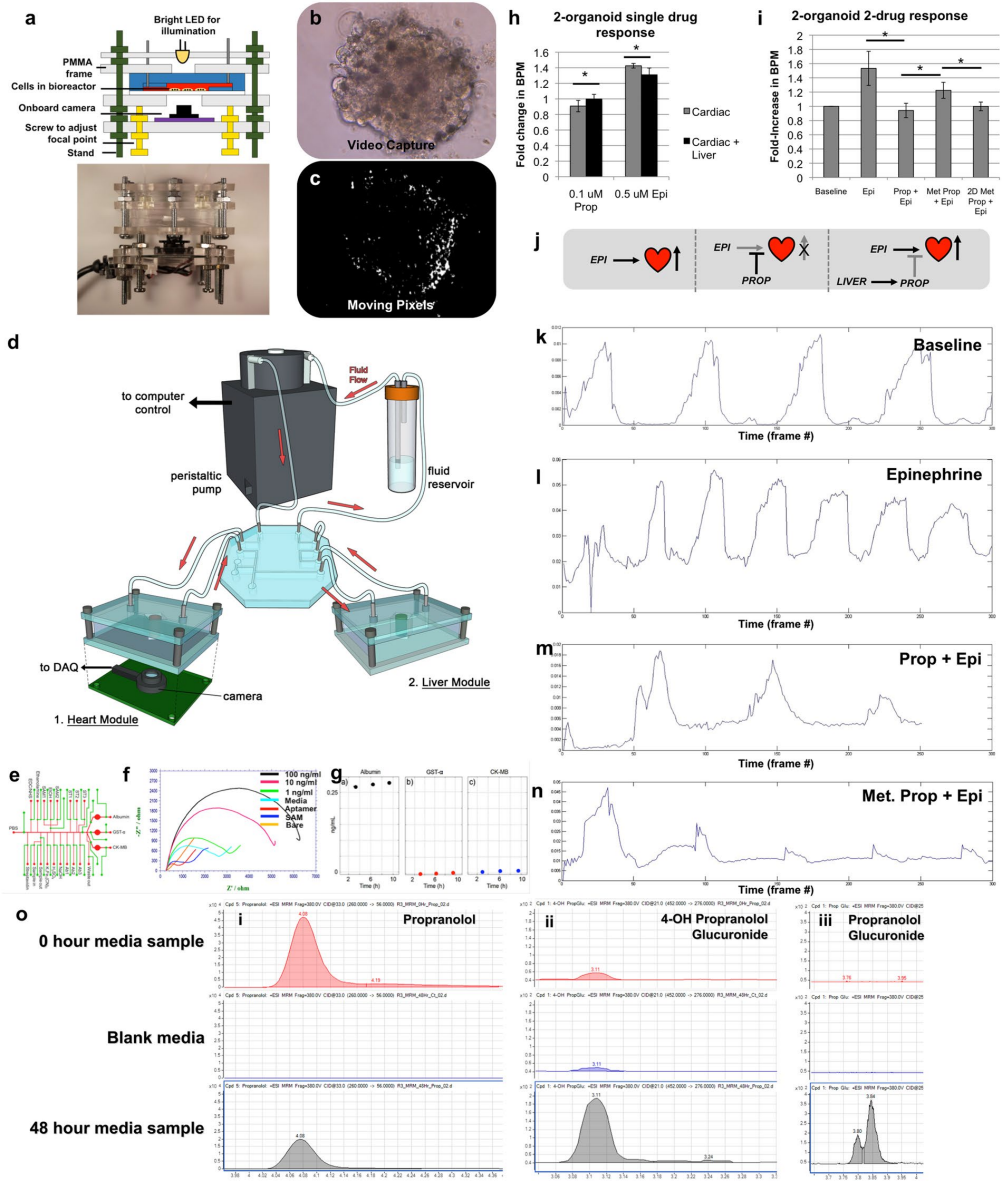
Two-organoid interaction: Combining liver and cardiac modules results in a biological system capable of an integrated response to drugs. (**a**) A depiction of the on-chip camera system used to capture real-time beating data of cardiac organoids during culture within the ECHO platform. (**b**) Screen capture from a video of a beating cardiac organoid within the microfluidic system, and (**c**) screen capture of thresholded pixel movement conversion of the beating cardiac organoid (generated by custom written MatLab code) allowing quantification of beat rates. (**d**) A depiction of the integrated liver and cardiac platform. (**e**) A depiction of the modular add-on electrochemical biosensing unit for quantifying albumin, α-glutathione-S-transferase, and creatine kinase by (**f**) monitoring increases in electrical impedance from biomarker deposition on the electrodes. (**g**) A 12-hour snapshot of electrochemical monitoring of the 3 aforementioned proteins under baseline conditions. (**h**) Incorporation of liver organoids results in an altered response of the cardiac organoids to both 0.1 μM propranolol and 0.5 μM epinephrine. (**i**) The effects of liver metabolic activity on downstream cardiac beating rates. BPM values increase from baseline with epinephrine 0.5 μM; Increases from epinephrine are blocked by 0.1 μM propranolol. When liver organoids are present and permitted to metabolically inactivate 0.1 μM propranolol, 0.1 μM epinephrine is capable of inducing an increased BPM value. Interestingly, if 2D cultured hepatocytes are substituted for the liver organoids, this effect is not observed, indicating that in 2D culture, hepatocyte drug metabolism is greatly reduced. Statistical significance: *p < 0.05. (**j**) Description of the 2 drug, 2 organoid interactions. (**k**–**n**) Cardiac organoid beat peak plots corresponding to the values presented in panel (i). (**o**) Relative quantification of propranolol and selected phase II metabolitesby triple quadrupole LC-MS MRM analysis. From the 0-hour sample and the 48-hour sample (i) propranolol decreases by factor three, (ii) phase II metabolites 4-OH propranolol glucuronide, and (iii) propranolol glucuronide increases. Blank media showed few if any detections of the compound. Results for 4-Hydoxypropranolol are not shown here, since it was not detected in any of the samples.

The cardiac module was developed next, since between liver and cardiac, since toxicities in these organs account for the majority of drug candidate failures and drug recalls^42^. These cardiac organoids demonstrated both normal cardiac biomarker expression and maintenance of long-term viability (Supplementary Fig. 4). After liver toxicity, cardiac toxicity is the largest source of drug candidate failure. Crucially, cardiac toxicity may not necessarily be manifest as cell death, but rather as changes in function; the most common of which is arrhythmia. Consequently, cardiac organoid beat rates served as one of the primary metrics for assessing cardiac organoid health. Visual monitoring was achieved using an onboard camera system^43^ that was customized to integrate with the cardiac construct microreactor housing (Fig. 3a) and allowed real time video capture of cardiac organoids (Fig. 3b, Movie S1). Using custom MatLab software (Data File S1–6), moving pixels in each frame were determined as a function of time, generating a visual representation of organoid contraction (Fig. 3c, Movie S2) as well as quantitative descriptions of beating rates (Supplementary Fig. 5a). Using this camera and software, physiologically-accurate response to commonly used cardio-responsive drugs isoproterenol and quinidine are described in Supplementary Fig. 5b,c.

Additionally, clinically-relevant concentrations of epinephrine and propranolol were assessed for their efficiency at inducing and preventing cardiac organoid beating rate increases. Epinephrine, an beta adrenergic receptor agonist causes increased heart rate, while propranolol, a broad beta blocker, can prevent beta adrenergic receptor agonist induced heart rate increases. Five epinephrine concentrations (0, 0.1, 0.5, 5, and 50 µM) were tested to determine the lowest concentration that initiates a clearly discernable increase in beating rate. Beating increased in a dose-dependent manner, reaching a plateau at 5 µM (Supplementary Fig. 5d). Next, four propranolol concentrations (0, 0.5, 5, and 20 µM) were administered to cardiac organoids. Organoids were incubated under these conditions for 20 minutes, after which epinephrine was added at 5 µM. In general, increasing concentrations of propranolol more effectively prevented epinephrine-induced increases in beating rates (Supplementary Fig. 5e), demonstrating an appropriate beta blocking response in the presence of epinephrine.

### Two-tissue liver- and heart-on-a-chip interactive screening

In the human body, organs interact with one another in complex ways. As an initial demonstration of multi-organoid interactions in our platform, we performed experiments in which the functionality of a cardiac construct was dependent on the metabolic capabilities of an upstream liver construct. The modular nature of the fluidic system was employed to produce such a system (Fig. 3d). Importantly, the relative ratio of liver to cardiac cells could be customized, thereby recapitulating a physiological ratio of liver-to-heart tissue mass. Specifically, a 5:1 ratio of liver-to-cardiac spheroids were employed within the two-tissue platforms.

From an analytical point of view, with the exception of the real-time integrated camera system (Fig. 3a)^43^, data output was collected in the form of discrete snapshots at a relatively small number of time-points using established, but tedious, traditional techniques such as ELISA and immunostaining. To improve on standard measurements, in-line sensors were combined with the microfluidic components in the form of an electrochemical sensor component. This sensor was designed to operate on the principle of impedance changes caused by antibody or aptamer binding^44^ providing constant measurements of up to three soluble biomarkers at a time (Fig. 3e–g). Figure 3g shows recorded electrochemical biomarker data for the 2-tissue platform over the course of a 12-hour cycle for tissue construct-secreted albumin, α-GST, and creatine kinase. Albumin levels were detectable and consistent, while α-GST and creatine kinase remained low, indicating no loss in viability under baseline.

Effects of epinephrine and propranolol were first tested independently in a cardiac-only system followed by testing in an integrated, dual organoid system. Drug concentrations of 0.1 µM propranolol and 0.5 µM epinephrine were chosen based on preliminary studies (Supplementary Fig. 5d,e). Treatment with propranolol resulted in a small (~10%), but significant (p < 0.05) decrease in beating rate in the cardiac-only system. However, in the presence of the liver construct, there was no decrease in beating rate, indicating metabolism of the drug (Fig. 3h). Similarly, treatment with epinephrine only resulted in a significant (~40%) increase in beating rate in the cardiac-only system. Addition of the liver component did not negate the epinephrine-induced beat rate increase, but reduced the effect to approximately a 30% beat rate increase (p < 0.05, Fig. 3i), further validating the integrated organoid system response.

Next, the interplay between both drugs in the cardiac-only and tandem systems was assessed using the same concentrations as above (overall experimental concept described in Fig. 3j). Propranolol was administered first and epinephrine was added subsequently. Depending on whether or not liver organoids were present, the effect of epinephrine was found to vary (Fig. 3i). Specific, beating plots for each condition are depicted in Fig. 3k-n. In Group 1 (cardiac organoids only), 0.1 µM propranolol remained active and successfully blocked the chronotropic effects of 0.5 µM epinephrine because there was no liver component to metabolize the blocking agent. In Group 2 (cardiac and liver components), a 25% increase in beat rate was observed after the epinephrine was administered, as compared to a 50% increase in cardiac beat rate in experimental controls where no propranolol was administered. This suggested that the 3D liver organoids were able to metabolize a sufficient amount of propranolol to allow epinephrine to induce a beat rate increase equivalent to approximately 50% of the control epinephrine-only response. This highlights the advantage that multiple organoid systems have compared to single organoid systems. Interestingly, conditions in Group 2 were repeated using a 2D hepatocyte culture comprised of 1–2 million cells on tissue culture plastic versus the 50,000 cells within the 3D organoids. The 2D cultures failed to achieve any restoration of the epinephrine-induced increase in beat rate (Fig. 3i), further demonstrating the lack of sufficient metabolic activity in 2D cultures compared to 3D systems. Example videos of these conditions are supplied as Movies S3–5.

For validation of the liver metabolism of propranolol, liquid chromatography-mass spectrometry (LC-MS) was performed using media aliquots from the experiments described above. Media was sampled at time points 0 and 48 h and relative quantification of propranolol and phase I and phase II metabolites were determined by LC-MS MRM and compound identities were confirmed by comparison to the retention times and MS/MS spectra of pure standards (Supplementary Fig. 6). At time point 48 h the propranolol concentration decreased by factor three (time point of interaction experiment). 4-OH propranolol as phase I metabolite has not been detected, but two phase II metabolites (4-OH propranolol glucuronide and propranolol glucuronide) which are not pharmacologically active. The results are shown in Fig. 3o. The measured decrease in propranolol concentration corresponds with the propranolol dose-inhibition curves and verifies that the shown organ-organ effect is caused by the decrease in propranolol concentration.

### Lung-on-a-chip

Following the 2-organoid interactive study above, the third tissue type to be developed for integration into the platform was lung. The lung serves as a unique point of entry for either toxic particles in the air or aerosolized drug compounds, and as such represents an important tissue for developing screening platforms^45^. As described above and shown in Fig. 1d, lung modules were fabricated using modified microreactor devices that contained a semi-permeable membrane on which layers of endothelial cells, lung fibroblasts, and lung epithelial cells were layered. The layered 3D organoid (Supplemental Fig. 7a) rapidly produced a cellular organization similar to that seen in native airway tissue, with a polarized epithelial surface exposed to the air-liquid interface, a stromal component providing tissue structure, and an endothelium forming a thin vascular barrier adjacent to the liquid media. Cross-sectional views of the structure show the 3 distinct cell populations using fluorescent probes, as well as H&E stained sections. Layered organoids can be maintained in culture for over 4 weeks (*not shown*) with long-term maintenance of cell viability. The advantages of the layered technique of organoid formation employed here are simplicity and the ability to rapidly establish an organized tissue representing the architecture of normal airway tissue. Importantly, the lung modules are compatible with transepithelial resistance (TEER) and short circuit current (Isc) electrophysiological sensing (Supplemental Fig. 7b), allowing straightforward monitoring of organoid integrity and changes in function such as ion channel activity, which is critical for lung function. In particular, the normal genetic coding of cystic fibrosis transmembrane conductance regulator (CFTR) chloride ion channel is required for normal lung function. Mutations in the gene can result in cystic fibrosis and dysregulation of epithelial fluid transport. Electrophysiological sensing of the 3D lung organoids confirmed that ion channels responded in a physiological manner to CFTR activation and inhibitory pharmaceuticals (forskolin and CFTRinh172) (Supplemental Fig. 7c,d). Additionally, as described for vascular TEER responses, histamine administration also resulted in a rapid change in TEER levels across the organoid (Supplemental Fig. 7e).

### Three-tissue liver, heart, and lung organ-on-a-chip

**Figure 4:**
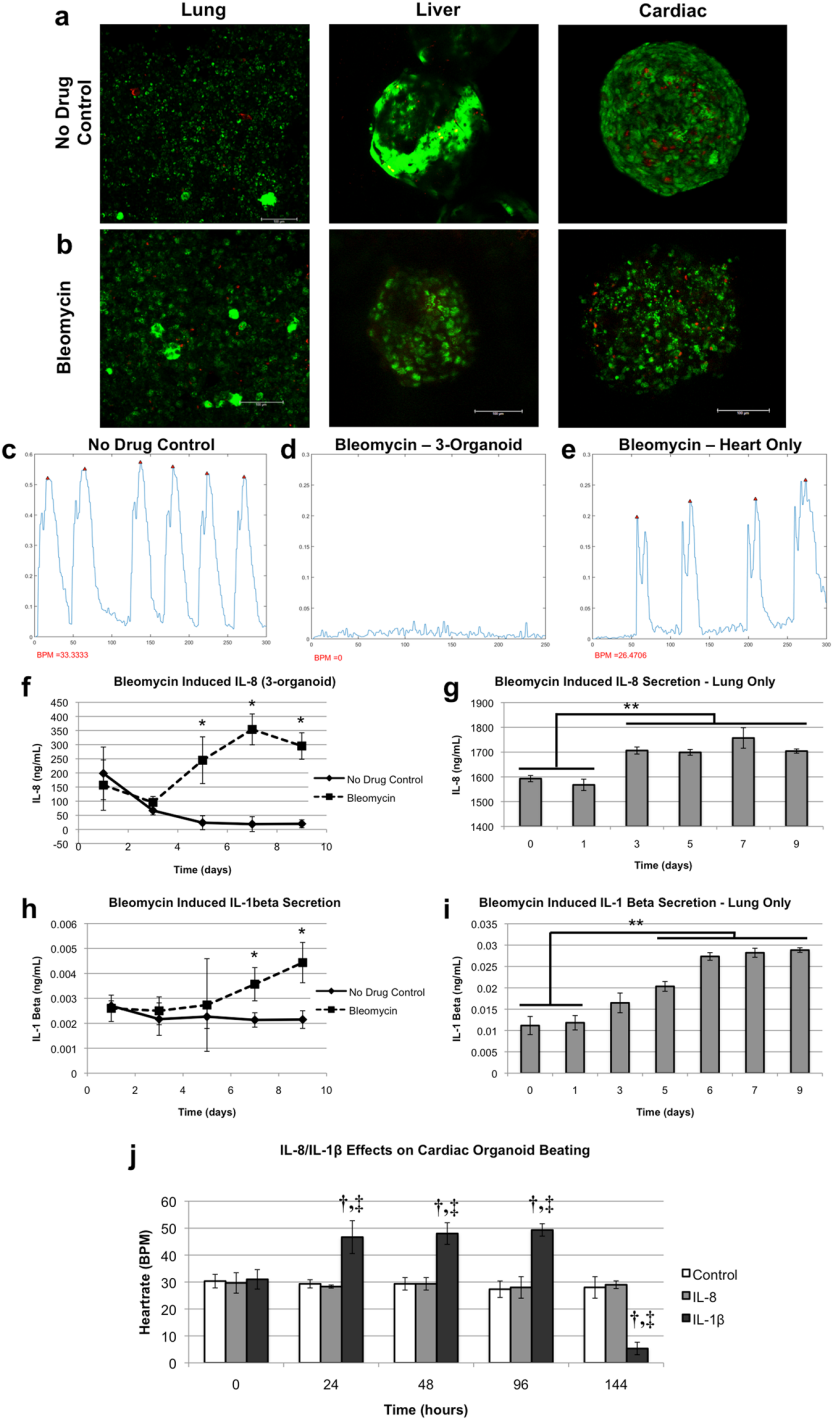
Three-organoid platform: Unanticipated side effect detection in a three organoid liver, heart, and lung system – Bleomycin induces lung inflammatory factor-driven cardiotoxicity. (**a**,**b**) Viability of lung, liver, and cardiac constructs under (**a**) a no drug control condition or (**b**) bleomycin after maintenance in a 3-organoid platform for 9 days. Bleomycin was added on day 3. Little cell death was observed, however, cardiac organoids in the bleomycin group appeared somewhat disaggregated. Green – Calcein AM-stained viable cells; Red – Ethidium homodimer-stained dead cells.(**c**–**e**) Cardiac organoid beating plots on day 9. In the 3-organoid platform cardiac organoids (**c**) beat steadily in the no drug control group, while (**d**) they had ceased beating completely under bleomycin treatment. (**e**) However, in systems containing isolated cardiac organoids, bleomycin did not cause a cessation of beating, suggesting an indirect bleomycin effect in the 3-organoid platforms. (**f**–**i**) Assessment of inflammatory factors following bleomycin administration on day 3. Interleukin-8, a factor commonly associated with lung inflammation was shown to increase over time in the (**f**) 3-organoid platforms, and (**g**) verified to be produced by lung constructs, but has not been linked to cardiotoxicity. However, interleukin-1β, an inflammatory factor that has been positively linked to cardiotoxicity, was also shown to increase over time in the (**h**) 3-organoid platforms, and (**i**) verified to be produced by lung constructs. (**j**) The effects of IL-8 and IL-1β on the cardiac organoids. IL-8 does not deviate from the control group, while IL-1β induces an initial increase, followed by a significant decrease in beating rate. Statistical significance in (**f**–**i**): *p < 0.05; **p < 0.01. Statistical significance in (**j**): ^**†**^p < 0.05 between indicated timepoint and 0-hour baseline; ^‡^p < 0.05 between the indicated IL-1β and both the control and IL-1β groups at the same time point.

In many instances during clinical trials of new drug candidates, and sometimes after the drug candidates have actually been approved for use, drugs fail due to unanticipated toxic side effects in tissues that are not directly targeted by the drugs themselves. These toxic side effects are critical information, which, if captured early in the drug development pipeline, could save both lives and millions or billions of dollars. Multi-organoid platforms have the potential to provide this information early in drug development. Lung-on-a-chip modules were then integrated into the platform with liver and cardiac microreactors under a common media (Fig. 1a). Perfusion was initiated for a 9-day time course, during which media aliquots were sampled every other day. The initial goal of this study was to use bleomycin to demonstrate a specific targeted insult to the lung, while not harming the liver or cardiac organoids. Bleomycin is a drug used against some cancers that causes significant lung fibrosis and inflammation, and as such, is commonly employed in a variety of lung-specific studies. Bleomycin was infused on day 3 of the study. On day 9 the study was concluded, and LIVE/DEAD analysis of organoids in both the no drug control groups and the bleomycin-treated groups yielded relatively low numbers of dead cells (Fig. 4a,b). However, we did observe a change in cardiac organoid morphology under bleomycin exposure with those cardiac organoids appearing less tightly aggregated. Additionally, while analysis of cardiac organoid beat rate showed consistent beating in the untreated platforms (Fig. 4c), the cardiac organoids in the bleomycin-treated systems ceased beating (Fig. 4d). This was an unanticipated side effect, as bleomycin is not known to cause cardiotoxicity. The study was then repeated using a cardiac-only system, in which it was shown that, indeed, bleomycin did not cause cessation of cardiac organoid beating (Fig. 4e). These results suggested that, rather than directly affecting the cardiac organoids, bleomycin may induce production of a secondary factor from one of the other tissues in the platform.

As bleomycin is known to induce upregulation of inflammatory product secretion in lung tissue and lung epithelium, lung inflammatory factors were assessed. First, media aliquots were probed for interleukin-8 (IL-8) by ELISA. As observed in the bleomycin-exposed lung, IL-8 levels were found to increase in the bleomycin-treated organoid systems (Fig. 4f) following the day 3 administration of bleomycin. Additionally, isolated lung modules treated with bleomycin showed the same increase in IL-8 (Fig. 4g), verifying secretion from the lung constructs. Similarly, the inflammatory marker interleukin-1β (IL-1β) has been shown to be secreted by lung epithelium upon bleomycin treatment. Again confirming established *in vivo* responses to bleomycin, in our organoid system quantification of IL-1β by ELISA showed increased levels towards the end of the study time course (Fig. 4h). Lung modules in isolation treated with bleomycin were also confirmed to secrete IL-1β (Fig. 4i). As IL-8 is not regarded as a cardiotoxic factor, our studies support the hypothesis that bleomycin-induced lung epithelium production of IL-1β contributed to inducing cardiotoxicity. Indeed IL-1β has been demonstrated to cause cardiomyocyte toxicity previously, an outcome that has been documented in the literature^46–49^, and may be a side effect of bleomycin administration in vivo^50–52^. To further assess this scenario – namely, that there were some cardiotoxic effects from IL-1β, and not IL-8, we performed the following experiment. Cardiac organoids were prepared and verified to have a consistent baseline beating rate throughout the batch. Human recombinant IL-8 or IL-1β were then added to the organoids in the cell culture media. In addition, control organoids received media with no added IL-8 or IL-1β. Over a time course of 6 days, videos of each organoid were taken at 4 time points (24, 48, 96, and 144 hours following initiation of the study), after which the videos were processed as described to yield beat kinetic plots. These data are summarized in Fig. 4j and representative beat plots are presented in Supplemental Fig. 8. These figures show that the cardiac organoids in the control and IL-8 experimental groups experience little if any significant changes in beating rate. Moreover, these experimental groups are not significantly different from one another at any of the 4 time points, suggesting that IL-8 has no apparent effect of organoid beating rates. Conversely, addition of IL-1β resulted in a significant increase in beating rate by approximately 60%. This increased heart rate endured through 96 hours. However, by 144 hours we observed a sharp decrease to levels significantly below that of the control and IL-8 groups. These results suggest that IL-1β has some influence in the beating kinetics of cardiomyocytes, as suggested above^46–49^. It is important to reiterate that cardiotoxicity is not typically attributed to the death of cells in the heart, but rather, in the case of many drugs that were recalled from the market due to cardiotoxicity, that toxicity takes the form of significant changes in beating kinetics, such as arrhythmias and other heartrate abnormalities. Importantly, these results demonstrate the detection of a toxic side effect that was not expected and would not be detectable without an integrated multi-organoid platform.

## Discussion

Development of new drug candidates has been limited and made incredibly expensive due to the failure of accurately modeling human tissues *in vitro*, while animal models allow only limited manipulation, are limited by costs, are generally low throughput, and are not necessarily predictive of results in humans. Traditionally, *in vitro* drug and toxicology testing has been performed using cell lines in 2D cultures. Despite yielding countless medical discoveries, 2D cultures fail to recapitulate the 3D microenvironment of *in vivo* tissues^4, 6, 7^. 3D systems composed of organoids or biofabricated tissue constructs have an increased capacity to respond accurately to drugs and toxins. Furthermore, recent advances of platforms that integrate multiple models of different tissue types seek to provide additional capabilities that take into consideration the complex interactions that can occur between different tissues in the body^29, 30^. Several groups have demonstrated the capability to maintain multi-tissue model system viability to up to 4 tissue types^29, 30^, mainly with organoids or tissue models in the form of 2D cultures or 3D cell lines. Our goal was to further advance these technologies by integrating more complex bioengineered tissues involving multiple cell types and ECM-based supportive biomaterials, in the same manner we have used to engineer tissues for replacement in patients, and to integrate these distinct tissue constructs – liver, cardiac, and lung – within a single recirculating perfusion system under a common media system (Fig. 1) with biosensing capabilities (Fig. 3e–g). We then describe applications of the components and combinations of the components of the platform to showcase the potential such a multi-tissue organ-on-a-chip platform may have. These examples include a liver insult and countermeasure study (Fig. 2), a demonstration of a two-organoid interdependent dual drug response (Fig. 3), a three-organoid demonstration of detection of an unanticipated drug side effect (Fig. 4), and a variety of individual organoid/tissue model characterization (Supplementary Figs 2–5 and 6), including histological and functional assays.

Importantly, differences were evident between what might be considered simple cell aggregates and the more complex 3D tissue organoids bioengineered in these experiments. The liver and cardiac organoids that we subsequently bioprinted into tissue-on-a-chip constructs far surpassed the functionality associated with simple cell aggregates. The term “cell aggregate” indicates a physical cluster of cells. Aggregates have been employed for many years, but traditionally do not necessarily have high tissue functionality. Furthermore, the customization we perform to provide a more supportive microenvironment provides additional characteristics to the system that have beneficial biological effects^36, 37^. Importantly, previous studies have demonstrated the utility of the liver-specific ECM-containing HA-gelatin hydrogel bioink at maintaining primary human hepatocyte cells and organoid viability and function *in vitro* ^36, 37, 40^. We originally applied an analogous bioink containing cardiac muscle ECM, but found that it did not promote sufficient cardiac organoid beating. Instead, a screening study of different bioprintable hydrogels found that a simple fibrin-gelatin bioink was a superior microenvironment for promoting cardiac organoid viability and function.

More important than individual organoid responses to drugs is a multi-organoid system response, in which the responses of one organoid have implications on the responses of other organoids, as occurs in actual human physiology. To explore this concept, individual liver and cardiac organoids were assembled into a dual organ system (Fig. 3). Since healthy liver can efficiently metabolize propranolol, rendering it ineffective at blocking cardiac beta-receptors, the effects of propranolol blocking and epinephrine-based beta-receptor activation was evaluated with and without liver organoids. In systems with no liver organoids, propranolol remained in its active form and successfully blocked epinephrine from inducing cardiac beating rate increases. However, when 3D liver organoids were introduced into the system, they metabolized the propranolol, and upon administration of epinephrine, beating rates increased, indicating significant inactivation of propranolol by the liver construct. Notably, when hepatocyte cultures in 2D were substituted for the 3D liver organoids, propranolol blocked epinephrine’s effects as if no liver cells were present at all, despite a 40X increase in cell number. Furthermore, mass spectrometry analysis confirmed liver metabolism of propranolol (Fig. 3o).

Additionally, a 3-tissue platform was prepared by integrating lung modules into the circulating perfusion system. As described in the results, the objective for this platform was to demonstrate the capability to specifically target only one tissue in the system with a drug, not all three tissues. However, this did not happen, but instead provided a potentially more valuable result. The initial intention was to induce lung toxicity using bleomycin, a well-documented drug that causes lung fibrosis, inflammation, and impaired lung function. However, the results described in Fig. 4 show a very different outcome, in which the cardiac organoids appeared to have suffered in terms of morphology, no longer appearing compact and healthy. Additionally, the cardiac organoids had completely ceased beating. Subsequent analyses suggested that bleomycin did not directly cause the cardiotoxicity, but rather, bleomycin caused the lung organoid to release inflammatory cytokines, including IL-1β, an inflammatory factor with known cardiotoxic properties, which then caused the observed toxicity in the cardiac organoids. While it is unclear whether this specific effect is present in humans that are given bleomycin, links between bleomycin and patient cardiac effects have been documented^50–52^. However, the results described here do highlight the ability of integrated systems to uncover potential negative drug effects for further evaluation during the preclinical phases of drug development.

Despite demonstration of these advanced integrated drug studies, there are still certain limitations to be addressed in continuing work. First, the system we describe here, but also most other organ-on-a-chip systems, are not at a stage where they can be easily deployed in high-throughput screening. This is perhaps the next significant hurdle in the development of these types of systems and adoption into practice by the pharmaceutical industry. Our team and others are currently working on techniques to 1) miniaturize these systems, and concurrently, 2) increase the level of throughput. We are translating the hardware from the devices described herein to new, inexpensive, and user-friendly manufacturing techniques. Second, while we describe several important drug treatment scenarios, we did not delve deeply into pharmacokinetics. We have recently begun deploying our system in the context of screening known toxic drugs and environmental toxins. As part of this effort we are working with our partners to more accurately describe the organoid-drug interactions in terms that better describe human-drug interactions. Third, three organoids are but a small representation of the many tissues and organs of the human body. Liver, heart, and lung are perhaps three of most important organs in terms of elucidating toxic responses to drugs and other compounds, yet we are currently working on other organoid and tissue construct models, which are being integrated with our three tissue platform. Eventually we expect to demonstrate the utility of a body-on-a-chip system containing many of the key functional organs in the human body.

Lastly, the system described here employs commercially available cells. Despite originally being sourced from patient tissues, we have treated these organoids and tissue constructs essentially as a generic liver, cardiac, and lung platform without significant consideration for personalized applications for specific patients. This is important, as we see these technologies having added value in personalized medicine. One could envision implementing organoids created from biopsied tissue from a patient’s organs that may be diseased or have a particular genetic profile that could influence drug-screening outcomes. Such screening studies could be incredibly useful for assessing efficacy of different potential treatments for that individual patient, thereby identifying the most effective, and just as importantly, the safest therapy to administer in the clinic. Moreover, collections of such organ-on-a-chip systems created from different patients would provide test platforms that better represent the heterogeneity in the human population, thereby improving overall drug development.

The work described here in demonstrates the creation of bioengineered tissue organoids that show functionality similar to native human organs. Further with sensor systems, paired with the physiologically accurate functional organoid technology, will generate extremely powerful research and diagnostic tools. The integration of multiple distinct bioengineered tissue models in single recirculating perfusion system comprised of liver, heart, and lung is also demonstrated. Importantly, the individual components of the platform respond appropriately to a panel of drugs, and even more importantly, when combinations of organoids are combined into a single platform, more complex integrated responses can be observed, where the functionality of one organoid influenced the response of another organoid. The integration of multiple functional human organoids and tissue constructs into single platforms under a common media results in additional capabilities that are impossible to observe in single tissue systems. This system has the potential for advanced *in vitro* drug and toxicology screening and disease modeling that may revolutionize the field of pharmaceutical development.

## Materials and Methods

### Experimental design

No animals or human subjects were employed in the studies performed. In general, experimental groups consisted of n = 6 or larger to provide robust statistical analysis. Often these experimental groups consisted of two actual experiments for validation of the data. Outliers were defined as data points two or more standard deviations from the statistical mean – however, since these were controlled *in vitro* studies, no outliers were observed. Time points for ending studies and ending data collection were defined prior to all experiments, and were not altered during the course of any experiments.

The objectives of the research were to develop a multi-tissue organ-on-a-chip platform comprised of purely primary or iPS cell-derived 3D organoids, using only commercially available cells, and then utilize this platform in drug screens. As our work progressed we realized that the more important objective was to demonstrate how important it is to integrate multiple organoids into a single system. Experiments were therefore designed that could elucidate the differences between single organoid systems and integrated organoid systems. Organoid systems were initiated using consistent techniques, after which they were randomly assigned to an experimental group. Since these were *in vitro* systems, not animals or human subjects, blinding was performed as best as possible, generally by keeping each organ-on-a-chip system labeled only with a number during the study and initial data analysis, after which the experimental group was revealed to pool individual system data sets.

### Liver and cardiac cell sources, culture, and organoid formation

All cells used were commercially sourced, human primary cells. Hepatic stellate cells (HSCs) (ScienCell, Carlsbad, CA) were expanded in culture for two passages before cryopreservation for use in organoid formation. During expansion, HSCs were cultured in 90% high glucose DMEM (Thermo Fisher, Waltham, MA) and 10% fetal bovine serum (Atlanta Biologicals, Flowery Branch, GA) on a rat tail collagen I coating (10 μg/cm^2^, Corning, Corning, NY) at 37 °C with 5% CO_2_. Primary human hepatocytes (Triangle Research Labs, RTP, NC) were thawed according to manufacturer instructions using Hepatocyte Thawing Medium (Triangle Research Labs). Kupffer cells were also thawed via manufacturer instructions (Gibco, Waltham, MA). Two-dimensional hepatocyte sandwich cultures were used as a comparison to the liver organoid. Primary human hepatocytes (Triangle Research Labs) were thawed as mentioned above, then plated on collagen coated (10 μg/cm2, Corning) 6-well culture plates, using Hepatocyte Plating medium (Triangle Research Labs) at a density of ~150,000 cells/cm^2^. Cells were incubated at 37 °C with 5% CO_2_ for 4 hours before adding matrigel as an overlay (BD Biosciences, San Jose, CA). Following further incubation for 24 hours, fresh HCM medium (Lonza, Walkersville, MD) was added.

Induced pluripotent stem cell-derived cardiomyocytes (iPSC CMs) were commercially sourced from Axiogenesis (cat. # COR.4U Cardiomyocytes). Human primary cardiac fibroblasts were commercially sourced from ScienCell (cat. # 6330). Prior to organoid formation, iPSC CMs were culture on tissue culture plastic for 48 hours in COR.4U medium until cells began beating spontaneously. At this point, iPSC CMs were harvested using trypsin-EDTA (Hyclone, Logan, UT).

Organoids were aggregated using GravityPlus hanging drop culture plates (inSphero AG). The cells were combined in a cell seeding mixture comprised of 90% HCM medium (Lonza), 10% heat-inactivated fetal bovine serum (Gibco), and rat tail collagen I (10 ng/µl, Corning). Liver organoids were produced with a mixture of 80% hepatocytes (Triangle Research Labs), 10% hepatic stellate cells (ScienCell), and 10% Kupffer cells (Gibco). Approximately 1500 cells per 40 μL media were used to form aggregates in hanging drop culture. Cardiac organoids were produced similarly. IPSC CMs were suspended in cardiomyocyte maintenance medium (CMM, Stem Cell Theranostics, Redwood City, CA). Fibroblasts were added as 10% of the total cell number, and the volume was adjusted to reach a cell density of 10,000 cells/mL. 100uL of cell suspension was pipetted into each well of a non-adherent, round-bottom, 96-well plates to produce spheroids (#7007, Corning) to result in approximately 1,000 cells/spheroid. Well plates were incubated and observed daily until spheroid formation, and then immediately used in experiments.

### Lung cell culture and module formation

The 3D airway organoids were modeled on the structure and cellular organization that are present in the airways. First, a polyester transwell membrane (Corning) was coated with lung ECM derived from decellularized lyophilized human donor lung tissue. Briefly, lung tissue ECM solutions were prepared as has been described previously for other types of tissue-derived ECM^36, 37^. Following preparation, transwell membranes were incubated with 100 μL of the ECM solution overnight at 37 °C, washed with PBS, and stored at 4 °C until seeding. Organoids were fabricated in three layers; Lower: lung microvasculature endothelial cells (Lonza CC-2527), Middle: airway stromal mesenchymal cells (donor derived), and Upper: bronchial epithelial cells (Lonza CC-2540S) cultured above the stroma. Specifically, airway stromal mesenchymal cells were seeded on one side of the membrane for 4 hours, after which they were washed and epithelial cells were seeded on top of the stromal cells for 4 hours. At this point, the device was flipped, and endothelial cells were seeded on the bottom side of the membrane for 4 hours. The layering approach rapidly produces a basic multicellular organization similar to that seen in native airway tissue, with an endothelium forming a vascular barrier exposed to liquid media, a stromal mesenchyme contributing to 3D tissue structure, and a polarized airway epithelium exposed to the air-liquid interface to stimulate cell polarization and maturation. Cells were maintained in Clonetics™ B-ALI™ air-liquid interface medium growth medium (Lonza) for 48 hours before performing air-lift and media change in the lower chamber with B-ALI™ differentiation medium. Media changes were performed every 48 hours.

### Hydrogel bioink preparation and bioprinting

Liver-specific hydrogel bioinks were formulated using a hyaluronic acid and gelatin hydrogel system infused with a liver ECM solution, containing growth factors, collagens, glycosaminoglycans, and elastin, which was prepared from decellularized porcine livers as described previously^36^. For bioink preparation, the thiolated hyaluronic acid and gelatin base material components from HyStem-HP hydrogel kits (Heprasil and Gelin-S, respectively, ESI-BIO, Alameda, CA) were dissolved in a 0.1% w/v solution of photoinitiator (4-(2-hydroxyethoxy)phenyl-(2-propyl)ketone, Sigma) to make 2% w/v solutions. A PEGDA crosslinker (MW 3.4 kDa, ESI-BIO) was dissolved in the phoinitiator solution to make a 4% w/v solution. Additionally, an 8-arm PEG Alkyne crosslinker (Creative PEGWorks, Winston-Salem, NC) was dissolved to make an 8% w/v solution. To prepare the hydrogel bioink solution, 4 parts 2% Heprasil, 4 parts 2% Gelin-S, 1 part crosslinker 1, 1 part crosslinker 2 is combined with 8 parts liver ECM solution and 2 parts Hepatocyte Culture Medium (HCM, Lonza). Unmodified HA (Vesta, Indianapolis, IN) and gelatin (300 bloom, Sigma-Aldrich, St. Louis, MO) was then supplemented to the bioinks (1.5 mg/mL and 30 mg/mL, respectively). The resulting mixture was vortexed to mix, transferred into a syringe or printer cartridge, and allowed to crosslink spontaneously for 30 minutes (stage 1 crosslinking). When secondary crosslinking (stage 2) was desired, for example, after bioprinting, the extruded stage 1-crosslinked gels were irradiated with ultraviolet light (365 nm, 18 w/cm^2^, BlueWave 75 UV Light Curing Spot Lamp, Dymax, Torrington, CT) to initiate a thiol-alkyne polymerization reaction.

Cardiac hydrogel bioinks were formulated using a simple fibrin-gelatin 2-part system. The first part was prepared by dissolving 30 mg/mL bovine fibrinogen (Sigma) and 35 mg/mL gelatin (Sigma) in PBS, while the second part was prepared by 20 U/mL thrombin (Sigma) in PBS. Crosslinking of the bioink components into a hydrogel was achieved by covering the desired volume of the fibrinogen-gelatin solution with the thrombin solution, thereby initiating enzymatic fibrinogen cleavage and subsequent crosslinking.

To fabricate liver constructs, primary liver spheroids were suspended within the hydrogel bioink solution, transferred to a bioprinter cartridge, after which the solution was allowed to undergo the first crosslinking stage (thiol-acrylate reaction) for 30 minutes. Following initial crosslinking, a 3D bioprinter developed in house^53^, was employed to extrude the hydrogel bioink concurrently with polycaprolactone (PCL, Sigma) to form a set of hydrogel “channels” between supportive PCL structures on top of a 7 mm by 5 mm diamond-shaped plastic coverslip. This architecture is described in Fig. 2b,c. Printing was performed under 20 kPa pressure applied by the bioprinter while the printhead moved in the X-Y plane at a velocity of approximately 300 mm/min. After deposition, administration of UV light for 1–2 seconds was used to initiate the secondary crosslinking mechanism, stabilizing the constructs and increasing material stiffness. Constructs were placed in the bottom of 12-well plates, covered with 2 mL HCM, and plates were placed in an incubator at 37 °C, 5% CO_2_ until further use.

To fabricate cardiac constructs, cardiac organoids were suspended within the fibrinogen-gelatin solution, and transferred to a bioprinter cartridge. The gelatin component added sufficient viscosity to the bioink, holding the organoids in suspension and facilitating smooth deposition. The 3D bioprinter deposited the organoid-laden bioink within a supporting PCL frame located along the perimeter of the same 7 mm by 5 mm plastic coverslips described above. Printing was performed as described above, after which the secondary solution of thrombin was used to cover the bioprinted construct, initiating crosslinking of the fibrinogen component. Constructs were placed in the bottom of 12-well plates, covered with 2 mL CMM with 20 μg/mL aprotinin (Sigma) to prevent enzymatic breakdown of the fibrin gel, and well plates were placed in an incubator at 37 °C, 5% CO_2_ until further use.

### Integration with microfluidic microreactor devices

Microfluidic devices were fabricated by assembly of polydimethylsiloxane (PDMS, Sylgard 184, Corning) components formed by conventional soft lithography and replica molding^39^. The micro-bioreactors consist of PDMS blocks to guide fluid flow that are held tightly from the top and bottom by PMMA (polymethyl methacrylate) clamps. The fabrication process started by machining two PMMA clamps that will secure the PDMS structures inside the bioreactor and will facilitate the addition of other structures. The PMMA layers were machined using laser cutting (5^th^ Gen Hobby Laser, Full Spectrum Laser, Las Vegas, NV) of 3 mm-thick PMMA (8560K239, McMaster). The bottom PMMA clamp had eight 2-mm holes on the edge of a 15 × 10 mm rectangle. The top part consisted of the same aligned eight holes (for screws clamping) and two 3.5 mm holes, with their centers aligned to the inlet/outlet ports of the micro-bioreactor.

The microfluidic components of the reactor were made using soft lithography from PDMS. To create the molds for the PDMS microfluidics components, PMMA sheets were machined using a laser cutter, or formed using SU-8 photoresist (MicroChem Corp., Westborough, MA). PDMS prepolymer was prepared by thoroughly mixing the silicone base and the curing agent (10:1 ratio by volume) for 5 min, followed by degassing of the PDMS mixture in a vacuum chamber for 30 minutes. Then, the pre-polymer was poured onto respective positive molds. For the thin lower layer (inlet piece) 2.0 g per 10-cm Petri dish was used, whereas 6.0 g was added for the thicker upper layer (outlet piece). A second degassing procedure was conducted to remove all the bubbles present, followed by curing of the PDMS at 80 °C for at least 90 minutes. Once cured, the two PDMS layers were cut against a mold. The cell chamber area was cut off from the lower layer, but saved for the plasma-bonding step later. Holes for inlet/outlet connections were cut using 1-mm punch on the upper layer.

Assembly of the system, described here recently^54^, started with the preparation of the bottom layer, which was performed using a standard irreversible air plasma bonding (Plasma Cleaner PDC-32G, Harrick Plasma) of the PDMS bottom layer to the TMSPMA-treated glass slide, such that the chamber faces opposite to the glass slide. Prior to bonding, the glass slide and PDMS layers were thoroughly cleaned against the scotch tape. Bonded constructs were then kept in the 80 °C oven for overnight.

The next step in the fabrication process of the bioreactor was the insertion of 1 mm connectors (Instech, Plymouth Meeting, PA) into the two punched holes of the top layer. A PMMA structure with corresponding holes was used as a protective layer to contain the PDMS in place near the connection. PDMS pre-polymer was added to completely fill the holes, followed by curing in 80 °C oven for 60 min. After curing, the connectors were carefully removed and PTFE tubing (ColeParmer, Vernon Hills, IL) was inserted into the holes and secured by epoxy glue. PDMS pads, which constitute the cushion layer, were prepared by pouring 7.5 grams of degassed PDMS into a 10 cm dish, followed by curing, to generate 1 mm thick PDMS pads. This cushion layer was used between the glass slide and the bottom PMMA cover. Before use, the layers of the microbioreactor are clamped and screwed to hold them together.

To accept bioprinted organoid constructs, the constructs on coverslips were transferred into the 7 mm by 7 mm organoid chambers micro-bioreactor devices using sterile forceps. Microreactors were then sealed and clamped immediately prior to use. Each device was connected by tubing to a microfluidic pump, bubble trap^55^, and media reservoir containing the appropriate media type depending on the subsequent experimental conditions (HCM, CMM, or a 50:50 common media). Flow was initiated at 10 µL/min and maintained to fill the system.

### Liver construct response to acetaminophen insult, and intervention with N-acetyl-L-cysteine

For initial assessment of the response of the liver-on-a-chip system to toxic drug insult, acetaminophen (APAP, Sigma) was employed. Liver organoids were cultured in microreactors as described before for 14 days. Media samples were collected on days 3, 6, 10, and 14. After media collection on day 6, 1 set of organoids continued with normal media, 1 set of organoids were treated with 1 mM APAP, and 1 set of organoids were treated with 10 mM APAP. To assess the effectiveness of a countermeasure treatment to be used in the liver organoid system, N-acetyl-L-cysteine (NAC, Sigma) was explored as a clinically relevant treatment against APAP-induced toxicity. This final set of organoids was treated with 10 mM APAP and 20 mM NAC. During media changes, groups receiving the drug treatment received fresh HCM also containing the appropriate drug concentration.

For assessment of liver-on-a-chip function, albumin and urea secretion under baseline conditions as well as during exposure to APAP, collected media aliquots were analyzed using a Human Albumin ELISA assay (Alpha Diagnostic International, San Antonio, TX) and the amount of secreted urea in the collected media was determined using a Urea colorimetric assay (BioAssay Systems, Hayward, CA). For viability assessment, organoids were removed immediately after the final media collection time point (day 14) for staining by LIVE/DEAD viability/cytotoxicity kits (Thermo Fisher). Staining consisted of incubation in 2 uM calcein AM (stains live cells green) and 4 uM EthD-1 (stains dead cells red) in a 1:1 PBS:HCM solution. Following staining, organoids were washed in PBS, fixed in 4% PFA, transferred to PBS, and imaged using macro-confocal microscopy (Leica TCS LSI, Leica Microsystems, Buffalo Grove, IL). Additionally, media samples were analyzed for presence of lactate dehydrogenase (LDH), an enzyme that is released from cells after toxicity causes cell membrane rupture, using a Lactate Dehydrogenase Assay Kit (Abcam, Cambridge, MA), and for α-GST, a hepatocyte-specific enzyme also released from cells after exposure to toxicity, using an α-GST Assay Kit (Oxford Biomedical Research, Rochester Hills, MI).

### Integrated liver and cardiac organoid system, biosensors, and integrated response to epinephrine and propranolol

To evaluate how the combination of liver and cardiac organoid types impact drug response, epinephrine (Sigma) and propranolol (Sigma) were tested independently and together. In the independent scenario, organoid platforms were prepared in two groups: Group 1 consisted of a set of organoids comprised only of cardiac, with “blank” liver modules. Group 2 consisted of both cardiac and liver. However, it should be noted that the individual cardiac and liver constructs were kept separate during the initial incubation period, while the drug was administered to the liver construct or “blank” liver module, after which the modules were joined for 30 minutes prior to cardiac beating rate assessment. Flow rates of 10 μL/min were used in all of the fluidic platforms. Baseline cardiac organoid beating rates were determined in each group prior to drug administration. Then, the drugs – either 0.1 μM propranolol or 0.5 μM epinephrine – were administered, allowed to incubate for 1 hour, after which the modules were joined, and data was collected.

To test the integrated response of the liver and cardiac system to epinephrine and propranolol combinations, the experimental groups described above were prepared and the same protocol (individual unit incubation prior to joining of modules) was followed. However, the incubation period was increased to overnight (18 hours). Both Group 1 and Group 2 were administered 0.1 µM propranolol. After the incubation period, the modules were combined and 0.5 µM epinephrine was administered to both groups. Additionally, in parallel, a Group 3 condition was employed, which mirrored Group 2, but used a 2D hepatocyte culture (1–2 million cells/well) instead of the liver construct as a 2D comparison. Detection of cardiac organoid beat rates was performed using the onboard camera system described above.

In order to detect biomarkers without a specific electrochemical reaction such as a mediator, electrochemical impedance spectroscopy (EIS) was employed as the measurement technique. EIS is an electrochemical technique that allows the investigation of the electrical properties of the electrode surfaces and binding kinetics of molecules between the electrolyte and the electrode surface. To capture biomarkers, antibodies or aptamers were used as the receptors to capture biomarkers, due to their selectivity and sensitivity against different antigens. A microelectrode was fabricated to perform electrochemical measurement^55^: the counter electrode and reference electrode, along with the working electrode, provide the circuit over which current is either applied or measured. Potassium ferricyanide (K3[Fe(CN)6]) electrolyte was added to the test solution to ensure sufficient conductivity. The combination of the electrolyte and specific working electrode material (Au) determines the range of the applied potential, and the attachment of antibodies to an electrode surface introduces a charge transfer resistance to the system. Electrochemical impedance spectroscopy (EIS) measurement was performed using a CHI 660E electrochemical workstation (CH Instruments).

The surfaces of the electrodes were functionalized by immobilizing streptavidin (SPV) on the working electrode through covalent bonding between the self-assembled monolayer (SAM) (carboxylic groups) and SPV (amine groups) by EDC/NHS (N-[3-dimethylaminopropyl]-N′-ethylcarbodiimide hydrochloride/N-hydroxysuccinimide). The SAM solution was prepared with mercaptoundercanoic acid in ethanol. The Au electrode was incubated within SAM solution and washed with ethanol. To create covalent linkers on the SAM layer, a 50 mM EDC/NHS mixture in citric acid (pH 4.5) was added on SAM functionalized electrodes for 15 min and dried to remove the excess EDC/NHS. Then the electrode was incubated in SPV. After washing, biotin functionalized antibodies were immobilized on the SPV functionalized electrodes. In case of aptamers, they were immobilized on the electrodes after the EDC/NHS step without SPV. All chemical reagents were purchased from Sigma Aldrich.

### Sample preparation and targeted LC-MS metabolic profiling of propranolol

For each individual sample, 500 ul of the media was added to a 3 kDa molecular weight cut-off filter spin column (Microcon YM-3 Centrifugal Filter, Millipore, Billerica, MA). Each sample was then centrifuged at 14,000 × g at 4 °C for 40 min. Following centrifugation, the flow-through was store at −80 °C until LC-MS analysis. Immediately prior to the experiments, samples were thawed and shaken vigorously for 30 s. During analysis, samples were maintained at 6 °C in a thermostatic auto sampler.

Propranolol, 4-hydroxy propranolol, propranolol glucuronide and 4-OH propranolol glucuronide standards were obtained from TLC Pharmaceutical Standards Ltd. Individual 1 µg/ml solution of each standard were prepared in methanol, then a mixed standard was prepared make a final concentration of 10 ng/ml.

LC-MS analysis were performed on an Agilent 6490 triple quadropole LC-MS/MS system with iFunnel and Jet-Stream^®^ technology (AgilentTechnologies, Santa Clara, CA) equipped with an Agilent 1260 infinity pump and autosampler. Chromatographic separation was performed on a Poroshell 120 EC-C18 column (50 mm × 3.0 mm i.d., 2.7 ìm particle size, Agilent, Santa Clara, CA). The LC parameters were as follows: autosampler temperature, 6 °C; injection volume, 4 ìl; column temperature, 35 °C; and flow rate, 0.4 ml/min. The solvents and optimized gradient conditions for LC were: Solvent A, water with 0.1% formic acid; Solvent B, 98% acetonitrile with 0.1% formic acid; elution gradient: 0–0.5 min 5% B; 5–8.5 min 95% B; post-run time for equilibration, 3 min in 5% B. MS was operated in positive-ion electrospray mode (unit resolution) with all analytes monitored by multiple reaction monitoring (MRM, Table 1). Compound identity was confirmed by comparison to the retention times of pure standards. The optimized operating ESI conditions were: gas temperature 230 °C (nitrogen); gas flow 11 L/min; nebulizer pressure 40 psi; sheath gas temperature 300 °C and sheath gas flow 10 L/min. Capillary voltages were optimized to 2500 V in positive mode with nozzle voltages of 1500 V. All data processing was performed with Agilent Mass Hunter Quantitative Analysis software package.

**Table 1:** Optimized triple quadrupole LC-MS parameters for MRM analysis.

Compound	Precursorion (m/z)	Production (m/z)	CAV	Collision energy (ev)
Propranolol	260	56	3	33
4-OH Propranolol	276	58	3	29
Propranolol Glucuronide	436	116	3	25
4-OH Propranolol Glucuronide	452	276	3	21

### Integrated liver, heart, and lung organoid system bleomycin screening

To generate three-tissue platforms, microreactors with liver organoids and cardiac organoids were connected in series with lung modules as shown in the schematic in Fig. 1a. Briefly, the pump drew media from the fluid reservoir and flowed it via the central routing breadboard to the liver, then the heart, followed by the lung, after which it circulated back to the reservoir. Flow rates of 10 μL/min and α-MEM (Hyclone, Logan, UT) with 10% FBS (Hyclone), 1% Pen/Strep (Thermo Fisher), and 1% L-glutamine (Thermo Fisher) were used in all of the subsequent fluidic platform studies. As additional controls, systems with only cardiac microreactors were prepared and subjected to the conditions described below.

After initiation of the three-tissue platforms, on day 3 bleomycin (10 μg/mL, Sigma) was added to half of the systems (n = 4), while the remaining half did not receive any drug treatment. Media aliquots were removed from the reservoir on days 1, 3, 5, 7, and 9. On day 9, cardiac organoids were assessed for beat rate analysis as described above, after which all organoids and constructs were assessed for viability by LIVE/DEAD and macro-confocal imaging as described above. Levels of IL-8 and IL-1β at each time point were assessed by ELISA (IL-8: ab46032, Abcam; IL-1β: ab46052, Abcam).

### Statistical methods

Data were expressed for each experimental group as mean ± SD and statistical significance determined using statistical analysis methods (GraphPad Prism, Graphpad Software Inc., USA, or Microsoft Excel, Microsoft Corporation, USA). One-way ANOVA were employed for multiple comparisons. Student’s t-tests were performed to compare the means of a normally distributed interval dependent variable for two independent groups. Confidence intervals of 95% or better were assumed to be significant.

## Electronic supplementary material

Movie S1

Movie S2

Movie S3

Movie S4

Movie S5

Supplemental Information
