# Supplementary material for: Multi-tissue interactions in an integrated three-tissue organ-on-a-chip platform
Source: Sci Rep. 2017 Aug 18;7:8837. doi: 10.1038/s41598-017-08879-x (PMC5562747; doi:10.1038/s41598-017-08879-x)
Supplement: Supplementary file 6 — Supplemental Information [file 41598_2017_8879_MOESM6_ESM.pdf]

Supplementary Materials:

Multi-tissue interactions in an integrated three-tissue organ-on-a-chip platform

Aleksander Skardal[1,2,*], Sean V. Murphy[1], Mahesh Devarasetty[1,2], Ivy Mead[1], Hyun-Wook Kang[1], Young-Joon Seol[1], Yu Shrike Zhang[3,4,5], Su-Ryon Shin[3,4,5], Liang Zhao[6], Julio Aleman[1,3,4,5], Adam R. Hall[1,2], Thomas D. Shupe[1], Andre Kleensang[6], Mehmet R. Dokmeci[3,4,5], Sang Jin Lee[1,2], John Jackson[1], James J. Yoo[1,2], Thomas Hartung[6,7], Ali Khademhosseini[3,4,5,8,9], Shay Soker[1,2], Colin E. Bishop[1], and Anthony Atala[1,2,*]

[1]Wake Forest Institute for Regenerative Medicine, Wake Forest School of Medicine, Medical Center Boulevard, Winston-Salem, NC, 27157, USA.

[2]Virginia Tech-Wake Forest School of Biomedical Engineering and Sciences, Wake Forest School of Medicine, Winston-Salem, North Carolina, USA

[3]Biomaterials Innovation Research Center, Division of Biomedical Engineering, Department of Medicine, Brigham and Women’s Hospital, Harvard Medical School, Cambridge, MA 02139, USA

[4]Harvard-MIT Division of Health Sciences and Technology, Massachusetts Institute of Technology, Cambridge, MA 02139, USA

[5]Wyss Institute for Biologically Inspired Engineering, Harvard University, Cambridge, MA 02139, USA

[6]Center for Alternatives to Animal Testing (CAAT), Bloomberg School of Public Health, Johns Hopkins University Baltimore, 615 N. Wolfe Street, Baltimore, MD, USA

[7]Steinbeis CAAT-Europe, University of Konstanz, Universitätstr. 10, Germany

[8]Department of Bioindustrial Technologies, College of Animal Bioscience and Technology, Konkuk University, Seoul 143-701, Republic of Korea

[9]Department of Physics, King Abdulaziz University, Jeddah 21569, Saudi Arabia

Supplementary Materials and Methods

Viability assays

Organoid viability was assessed by ATP production as a measure of metabolic activity. CellTiter-Glo assay (Promega) was used to measure ATP by transferring one organoid/well to a black, opaque 96-well plate (Corning). Blanks were included using HCM medium (Lonza) at 80 µl/well. 80 µl of prepared CellTiter-Glo buffer was added per well and plate was placed on shaker for 5 minutes to lyse cells, then further incubated for 15 minutes protected from light. Plate was read using plate reader (SpectraMax M5, Molecular Devices) with an integration time of 0.5 sec/well. Sample time points were compared via two-sample unequal variance t-test. Live/dead stain was also used to assess viability. Organoids were washed in PBS and then stained with LIVE/DEAD viability/cytotoxicity kit (Life Technologies): 2µL/mL ethidium homodimer-1 and 0.5µL/mL calcein AM (diluted in PBS) for 45 minutes at room temperature, protected from light. Organoids were transferred to a depression glass slide (Erie Scientific) and then imaged using TCS LSI macro confocal microscope with 5x macro objective (Leica).

Immunohistochemistry

Organoids were collected and fixed in 4% paraformaldehyde for 1 hour at room temperature. Organoids were embedded in Histogel (Richard-Allan Scientific) and then dehydrated with a series of graded ethanol washes before paraffin embedding to be sectioned at 4µm. Sections were stained with hematoxylin and eosin and imaged via light microscopy using a DM4000B microscope (Leica).

All washes were performed in TBS buffer and incubation steps at room temperature unless otherwise stated. Sections were deparaffinized and hydrated to water and then a heat induced epitope retrieval step was performed in 0.01M citrate buffer (pH 6.0). Endogenous enzyme activity was blocked using Duel Endogenous Enzyme Block (Dako) incubated for 10 minutes. Slides were blocked in Serum Free Protein Block (Dako) for 15 minutes. Primary antibodies were diluted in Antibody Diluent (Dako) and incubated overnight at 4ºC. Antibodies used include: mouse anti-human serum albumin (Abcam, ab10241), rabbit anti-cytokeratin 18 (Abcam, ab52948), rabbit anti-cytochrome P450 reductase (Abcam, ab13513), rabbit anti-GFAP (Abcam, ab7260), rabbit anti-connexin 32 (Invitrogen, 71-0700), and rabbit anti-E-cadherin (Abcam, ab40772), mouse anti-troponin T-C (Santa-Cruz, sc73234). Secondary antibodies were diluted in Antibody Diluent (Dako) and incubated for 1 hour. Secondary antibodies used include: peroxidase AffiniPure donkey anti-rabbit IgG (Jackson ImmunoResearch Labs, 711-035-152), biotin anti-mouse IgG (Vector Labs, BA-2000) and biotin anti-rabbit IgG (Vector Labs, BA-1000). For HRP conjugated antibodies, samples were developed using the NovaRed substrate kit (Vector). For avidin-biotinylated conjugate antibodies, slides were developed using Vectastain Universal ABC-AP kit (Vector) and VectorRed AP substrate (Vector). Slides were stained with hematoxylin and then permanently coverslipped with Mounting Media 24 (Leica). Slides were imaged via light microscopy using DM4000B microscope (Leica).

Whole mount organoid immunofluorescence analysis of cardiac organoids

Cardiac organoids were analyzed via whole mount immunofluorescence imaging. All washes were performed with PBS and steps were performed at room temperature unless otherwise stated. Organoids were collected and fixed with 4% paraformaldehyde, incubated for one hour on shaker. Organoids were permeabilized using 0.5% Triton-X 100, incubated for one hour on shaker. Samples were treated with Protein Block (Dako) for one hour. Primary antibodies were diluted in Antibody Diluent (Dako) and incubated overnight at 4ºC. Primary antibodies used include: rabbit anti-VEGF (Santa Cruz, sc-152), mouse anti-a-actinin (Santa-Cruz, sc-17829), and mouse anti-MYL7 (Santa-Cruz, sc-365255). Secondary antibodies were diluted in Antibody Diluent (Dako) and incubated overnight at 4ºC. Secondary antibodies used were: goat anti-rabbit AF488 (Life Technologies) and goat anti-mouse AF594. Samples were stained with DAPI for 20 minutes on shaker. Samples were transferred to a depression glass slide (Erie Scientific) for imaging using TCS LSI macro confocal with 5x macro objective (Leica).

Liver organoid characterization

Urea and albumin production were measured by collecting supernatant from individual wells 24 hours following medium change. Urea production was measured using a colorimetric assay, Quantichrom Urea Assay Kit, (BioAssay Systems) following manufacturer's instructions. Samples were measured in a 96-well clear assay plate (Corning) using plate reader set to 430nm (SpectraMax M5, Molecular Devices). Data were analyzed using two-sample unequal variance t-test. Albumin production was measured using Human Albumin ELISA kit (Alpha Diagnostic International) according to manufacturer's instructions. Samples were measured using plate reader set to 450nm (SpectraMax M5, Molecular Devices) and data were analyzed using two-sample unequal variance t-test.

All drug compounds used for this experiment were sourced from Sigma Aldrich. Drug toxicity in the organoids and monolayer cultures was assessed by inducing cytochrome P450 activity using a mixture of rifampicin (25mM), 3-methylcholanthrene (3.78μg/mL), and phenobarbital (58.0μg/mL) in HCM medium (Lonza), inducing the cells for 24 hours. Then diazepam was added (2.5μg/mL) in HCM medium for 24 hours. Diazepam metabolites temazepam, nordiazepam, and oxazepam were measured in the cell supernatant. Sample volumes were measured with 4-OH coumarin added as an internal standard to a final concentration of 500pg/μl, and 25μl injected onto a Phenomenex Hypersil 3μm C18-BD 150mm length X 2mm I.D. column (P/N 00F-4018-B0), maintained at 50°C and eluted at a flow rate of 0.2ml/min. The LC gradient was as follows: 95% A at 0min., to 30% A from 0-6min., hold at 30% A from 6-20min., to 95% A from 20-22min., hold at 95% A from 22-30min, where solvent A was 95:5 (v/v) H2O:Methanol + 0.15% formic acid, and solvent B was methanol + 0.15% formic acid. The system used was a Thermo-Scientific Quantum Discovery Max triple quadrupole mass spectrometer run in positive ion and multiple reaction monitoring modes, automated by a Spark Holland LC, and a Reliance auto-sampler and conditioned stacker maintained at 4°C. The spray voltage was 3500V, the capillary temperature was

250°C, the scan time was 0.1 seconds, the Q1 and Q3 peak widths were both 0.70, and the Q2 collision gas pressure was 0.8 mtorr.

For phenotype characterization via immunostaining, organoids were maintained in culture for up to 28 days, during which several analyses were performed at various time points. Spent media was replaced with fresh HCM on day 3, day 6, 10, 14, 17, 21, 24, and 28. After 8 days, organoid constructs were fixed in 4% PFA and rinsed in PBS, after which constructs were maintained in PBS at 4°C until processing for histological analysis (described below). For albumin and urea secretion analysis organoids were maintained in culture for 14 days, during which media was collected and replaced with fresh HCM on days 3, 7, 10, and 14. For viability assessment, organoids were maintained in culture for up to 28 days. Subsets of organoids were removed from microreactor culture on day 1, day 14, and day 28 for staining by LIVE/DEAD viability/cytotoxicity kits (Life Technologies), after which they were fixed in 4% PFA, transferred to PBS, and imaged using macro-confocal microscopy (Leica TCS LSI)

Cardiac organoid characterization

The onboard camera was designed and fabricated based on a commercial cost effective webcam (Logitech C160) and significantly improved from lens-free versions. The schematic in **Fig. 3a** shows the fabrication procedure of the microscope with parts assembled from a webcam. First the cover of the webcam is disassembled to retrieve the CMOS imaging sensor. The lens of the webcam is then detached from its initial location, flipped, and integrated back to the holder to convert it into a magnifying lens. A base was then constructed for the mini-microscope to fit onto the bottom of the bioreactors. The base consisted of a dual-layer structure of PMMA sheets (1/8" Thick, 12" x 12", McMaster 8505K11) cut into the dimensions of the bioreactors using a laser cutter (VLS 2.30 Desktop Laser System, Universal Laser Systems). Using 4 sets of screw/bolts, the CMOS module was tightly clamped in between a pair of PMMA structures. Additional 4 sets of screw/bolts were further mounted at the corners of the structures to function as the focus knobs. Only very minor alteration to the bioreactor itself was needed, i.e., 4 extra holes were drilled on the lower PMMA board to fit the imager at the bottom.

During culture of cardiac constructs, videos were captured to analyze cardiac organoid beating rates. Video files were analyzed using custom written MatLab code (Data File S1) with a series of MatLab functions (Data Files S2-6). The software created a reference frame, based on the first frame of the video, and compared pixels in each subsequent frame, determining which pixels represented movement over time. The moving pixels in each frame were then used to generate a black and white pixelated representation of beating behavior, allowing visualization of beat propagation, and generation a plot showing the number of moving pixels versus time, allowing determination of beating rates.

To assess cardiac organoid beating rate response to drugs, videos of cardiac organoids were captured under baseline conditions or having been treated with 0.1 mM isoproterenol, 1 μM quinidine, or combinations of epinephrine and propranolol. For the latter two drugs first, epinephrine was administered at the following concentrations and

organoid beating rates were determined: 0 μM, 0.1 μM, 1 μM, 10 μM, 50 μM. Next, the response of epinephrine under the influence of propranolol, a beta-blocker that prevents increases in heart rate *in vivo*, was assessed by initial incubation of cardiac organoids with 0 μM, 0.5 μM, 5 μM, and 20 μM for 15 minutes, after which epinephrine was administered at a concentration of 5 uM, and beating rate was determined visually under the microscope.

Lung module characterization

Lung airway organoid were characterized using a combination of morphholological assessment and function via TEER sensing. The 3 individual cell populations employed in fabricating the lung organoids were labeled prior to organoid fabrication: Airway epithelial cells were labeled using DiO fluorescent probes (Thermo Fisher); Airway stromal cells were labeled using DiI fluorescent probes (Thermo Fisher); and lung vascular endothelial cells were labeled using DiI fluorescent probes (Thermo Fisher). Cross-sectional visualization of the 3 cell population layers within the organoids was achieved by both H&E staining and confocal imaging of organoids that had been formed using fluorescently tagged cells. Functional assessment was achieved by Isc and TEER sensing, particularly focusing on CFTR chloride ion channel activity. Changes CFTR ion channel Isc were measured in response to several CFTR activating or inhibitory pharmaceuticals. Specifically, forskolin (Sigma), a labdane diterpene chloride channel activator, CFTRinh172, a CFTR inhibitory molecule (Sigma), and UTP (uridine triphosphate nucleotide, Sigma). Additionally, TEER levels were measured in response to histamine, which is known to cause permeability in endothelium.

Cardiac organoid treatment with IL-8 and IL-1β

Cardiac organoids were maintained in CMM (Stem Cell Theranostics) as described in the main text. Cell culture media solutions were prepared in 3 experimental groups: normal CMM (control condition), CMM with IL-8 (1.7 ng/mL, ab9631, abcam), and CMM with IL-1β (0.03 ng/mL, ab9617, abcam). Concentrations of the IL solutions were based on data in the lung-only bleomycin insult studies shown in **Fig. 4**. Original CMM aliquots were removed from round bottom well plates containing cardiac organoids and replaced with the 3 media conditions above (**n = 3** for each condition). Videos of each organoid were captured at 5 time points (prior to media change, and 24, 48, 96, and 144 hours following initiation of the study), after which the videos were processed by the MatLab code described above to yield organoid beat plots.

Supplementary Results (Basic organoid characterization)

Liver organoid characterization

Liver organoids were produced using the hanging drop culture method that consistently formed uniform spheroidal aggregates of ~250μm in diameter. Liver organoids, comprised of human hepatocytes, stellate cells, and Kupffer cells, remained stable (+/- 10μm) throughout the 28 day culture period (**Supplementary Fig. 2a**),

maintaining a size, dictated by cell number, that balances biological function with solute perfusion constraints that can cause hypoxia and the formation of a necrotic core. Hematoxylin and eosin (H&E) staining (**Supplementary Fig. 2d**) shows a compact organoid structure with cell types of different morphologies distributed throughout the organoid. Appropriate hepatocyte markers were analyzed by staining for albumin (**Supplementary Fig. 2e**) and cytochrome P450 reductase (**Supplementary Fig. 2h**), showing the expected expression pattern. Connexin 32, a hepatocyte specific marker, was visible throughout the structure (**Supplementary Fig. 2g**). E-cadherin (**Supplementary Fig. 2f**), a cell-cell adherence protein, was expressed by hepatocytes, demonstrating the formation of cell connections important for long-term maintenance of cell differentiation. Glial fibrillary acidic protein (GFAP, **Supplementary Fig. 2i**), a marker for hepatic stellate cells, was only found in a few regions, congruent with desired proportions. CD68 staining (**Supplementary Fig. 2j**) revealed the presence of Kupffer cells.

Liver organoids maintain consistent metabolic levels, measured ATP quantification at multiple time points over 28 days in culture (**Supplementary Fig. 2b**). LIVE/DEAD staining provided similar evidence of viability (**Supplementary Fig. 2c**). Additional functionality was assessed by measuring urea and albumin production over time. Secretion of these molecules was maintained for at least 28 days in culture, confirming long-term hepatocyte viability and functionality (**Supplementary Fig. 3a-b**). Notably, organoids produced significantly more urea and albumin than traditional monolayer cultures, despite containing fewer cells per culture (Liver organoid: ~$1.5X10^3$ cells/sample. Monolayer cultures: ~$1.5X10^6$ cells/sample). Monolayer cultures also failed to maintain measurable urea and albumin production after 21 and 14 days of culture, respectively.

To evaluate drug metabolism, cytochrome P450 enzymes were induced using rifampicin, 3-methylcholanthrene, and phenobarbital. Subsequently, organoids were exposed to diazepam, which is converted into primary metabolites temazepam and nordiazepam primarily by CYP3A4 and CYP2C19, and a secondary metabolite, oxazepam (**Supplementary Fig. 3f**). Liver organoids were found to have measurable cytochrome P450 drug metabolism activity for at least 28 days, in comparison to standard monolayer sandwich cultures that lost CYP450 activity after 7 days (**Supplementary Fig. 3c-e**). It is also important to note again the difference in total cell number between the 3D culture model (~$1.5X10^3$ cells/sample) and the 2D culture model (~~$1.5X10^6$ cells/sample).

Cardiac organoid characterization

Like the liver organoids, cardiac organoids were also produced using the hanging drop culture method that consistently formed uniform spheroidal aggregates of ~250µm in diameter. Cardiac organoids positively expressed VEGF (**Supplementary Fig. 4a**), which is expressed in 3D cardiomyocytes cultures, but not 2D cultures, suggesting improved capability to induce neovascularization. Actinin (**Supplementary Fig. 4b**), a microfilament protein required for attachment of actin to Z-lines of cardiac myofibrils, and cardiac troponin-T (**Supplementary Fig. 4c**), a protein essential for cardiac muscle contraction, were also strongly expressed. Organoids had very low levels of myosin regulatory light chain 7 expression (MYL7, **Supplementary Fig. 4d**), which if expressed at higher levels would indicate regression of the cardiomyocytes to an immature state.

Interestingly, expression of MYL7 was only observed in node-like regions on the perimeter of the organoids suggesting that these regions may be the point of beat initiation, as immature cardiomyocytes maintain the potential for spontaneous beating. H&E staining showed a consistent distribution of cells throughout the interior of the organoids, as well as more diffuse aggregation compared to liver organoids (**Supplementary Fig. 4e**). Live/dead staining over various time points in culture demonstrated high levels of viability (>95%) on day 1, day 28, and day 35 of culture (**Supplementary Fig. 4f-h**).

Physiologically-accurate response to drugs and other stimuli is a necessary feature of engineered cardiac constructs. In order to test this capability in our system, a variety of drugs known to modulate beating kinetics were administered to the cardiac constructs under visual inspection. For example, isoproterenol is known to have a positive chronotropic effect induced by its beta-adrenergic agonism. Indeed, exposure of our cardiac organoids to 0.1 mM isoproterenol was found to increase organoid beating rate (**Supplementary Fig. 5b**). Conversely, quinidine is a negative chronotropic agent that slows action potential propegation by blocking sodium channel activity. Likewise, exposure of cardiac organoids to 1 μM quinidine also slowed organoid beating rate (**Supplementary Fig. 5c**).

Lung module characterization

3D lung organoids were formed using a combination of airway epithelial cells, airway stromal cells, and lung microvascular endothelial cells. The layered 3D organoid (**Supplemental Fig. 6a**) rapidly produced a cellular organization similar to that seen in native airway tissue, with a polarized epithelial surface exposed to the air-liquid interface, a stromal component providing tissue structure, and an endothelium forming a thin vascular barrier exposed to liquid media. Shown within the layered system are the airway epithelial cells, labeled with DiO, the airway stromal cells, stained with DiI, and the lung vascular endothelial cells, stained with DiD. Cross-sectional views of the structure shown the 3 distinct cell populations using fluorescent probes, as well as H&E stained sections. Layered organoids can be maintained in culture for over 4 weeks (*not shown*) with maintenance of transepithelial resistance and cell viability. The advantage of the layered technique of organoid formation employed here is simplicity, and the ability to rapidly establish an organized tissue representing the architecture of normal airway tissue. Importantly, like with the vascular constructs described above, the lung modules are compatible with Isc (short circuit current) and TEER sensing (trans-epithelial electrical resistance, **Supplemental Fig. 6b**), allowing straightforward monitoring of organoid integrity and changes in function such as ion channel activity, which is important in the lung. In particular, the normal genetic coding of CFTR (cystic fibrosis transmembrane conductance regulator) choride ion channel is required for normal lung function. Mutations in the gene can result in cystic fibrosis and dysregulation of epithelial fluid transport. TEER sensing of the 3D lung organoids showed that the ion channels responded in a physiological manner to CFTR activating or inhibitory pharmaceuticals (forskolin, CFTRinh172, and UTP, **Supplemental Fig. 6c-d**). Additionally, as described for vascular TEER responses, histamine administration also resulted in a rapid change in TEER levels across the organoid (**Supplemental Fig. 6e**).

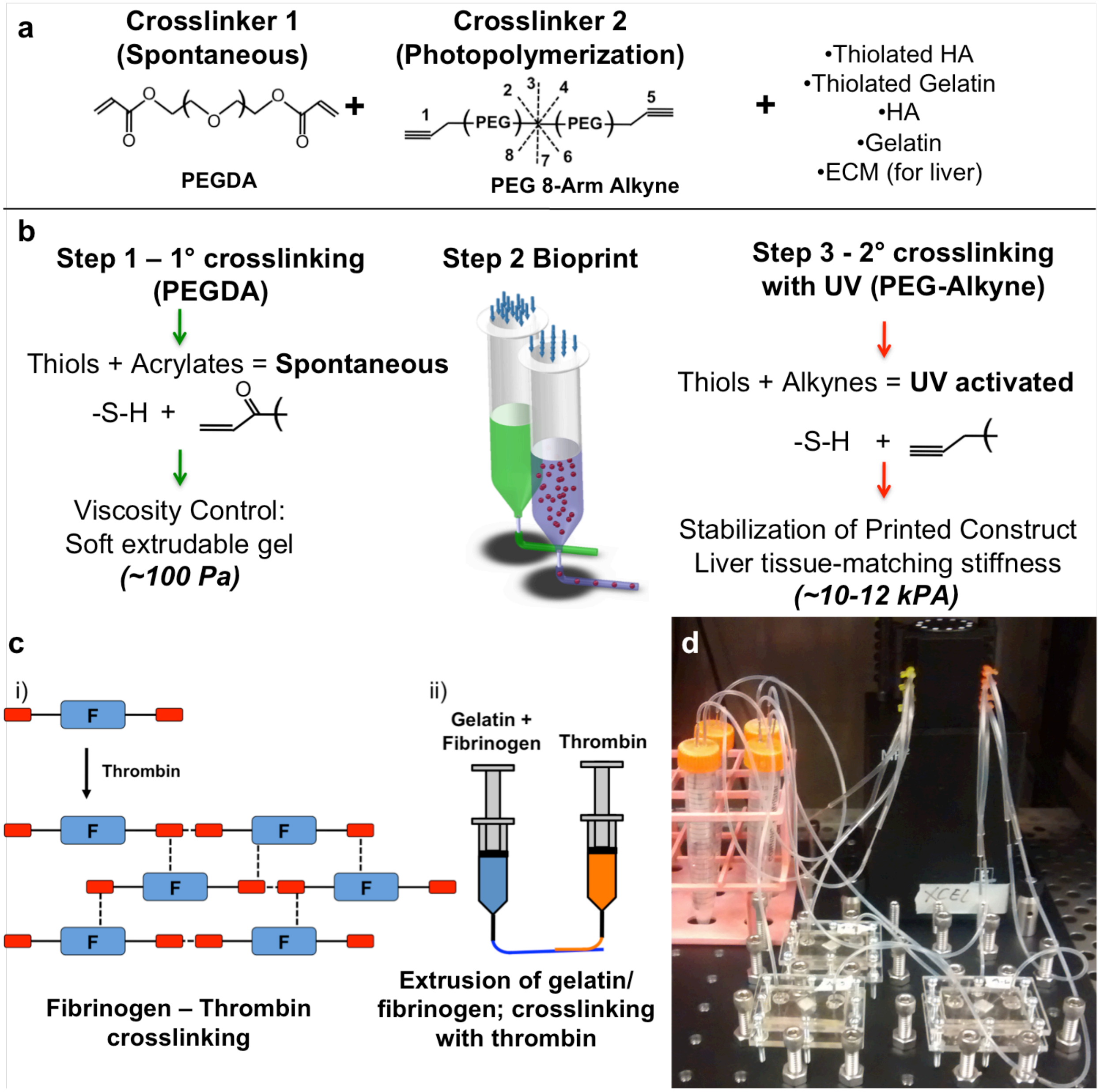

Supplementary Figure 1. Bioprinting of hydrogel bioinks strategy, fluidic system overview. a-b) Liver bioink formation and implementation. a) Strategy of formulation of printable bioinks comprised of acrylate-based crosslinkers (crosslinker 1), alkyne-based crosslinkers (crosslinker 2), thiolated HA, thiolated gelatin, and unmodified HA and gelatin. b) Implementation of bioprintable hydrogel bioinks. The bioink formulation is prepared and spontaneously crosslinks through thiol-acrylate binding, resulting in a soft, extrudable material. Bioprinting is performed. Lastly, the bioprinted structures are fused, stabilized, and brought to the target stiffness. c) Cardiac bioink formation and implementation. d) An operational three organoid-on-a-chip fluidic system. Three fluid circuits support three organoid devices, a parallel circuit microfluidic pump, media reservoirs, and bubble traps (not visible).

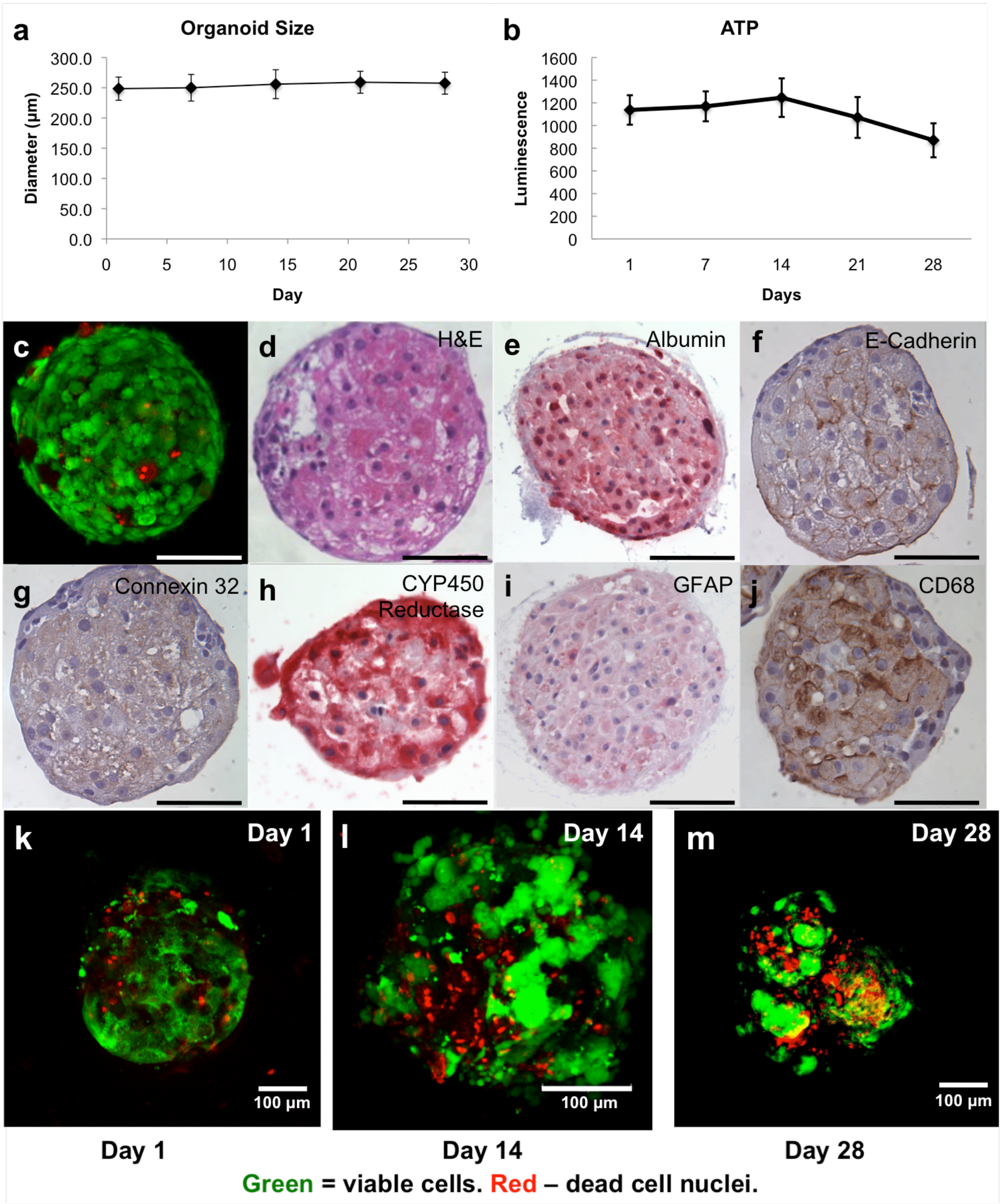

Supplementary Figure 2. Liver organoids exhibit liver-specific markers and remain stable and viable long term. a) Average organoid diameter remains consistent over 28 days. b) Liver organoids remain metabolically active over 28 days, as determined by luminescence readings of ATPase. c) LIVE/DEAD staining (shown at 14 days) shows high cell viability in the organoids. Green – calcein AM-stained viable cells; Red – ethidium homodimer-1-stained dead cells; Diameter 261μm. d-j) Histological and

immunohistochemical staining depict organoid structure and organization. d) H&E staining shows overall organoid morphology. Primary human hepatocytes are identified by e) albumin expression, and exhibit epithelial organization shown through f) E-cadherin expression around the cell membrane, and also express g) connexin 32 and h) cytochrome P450 reductase. Hepatic Stellate and Kupffer cells are identified by i) GFAP, and j) CD68, respectively. Purple – hemotoxylin-stained nuclei; Pink – cell cytoplasm; Brown – indicated stain; Scale bar – 100 μm. k-m) Viability of liver organoids following integration into microreactor devices and culture under circulating perfusion for k) 24 hours, l) 14 days, and m) 28 days. Green – calcein AM-stained viable cells; Red – ethidium homodimer-1-stained dead cells.

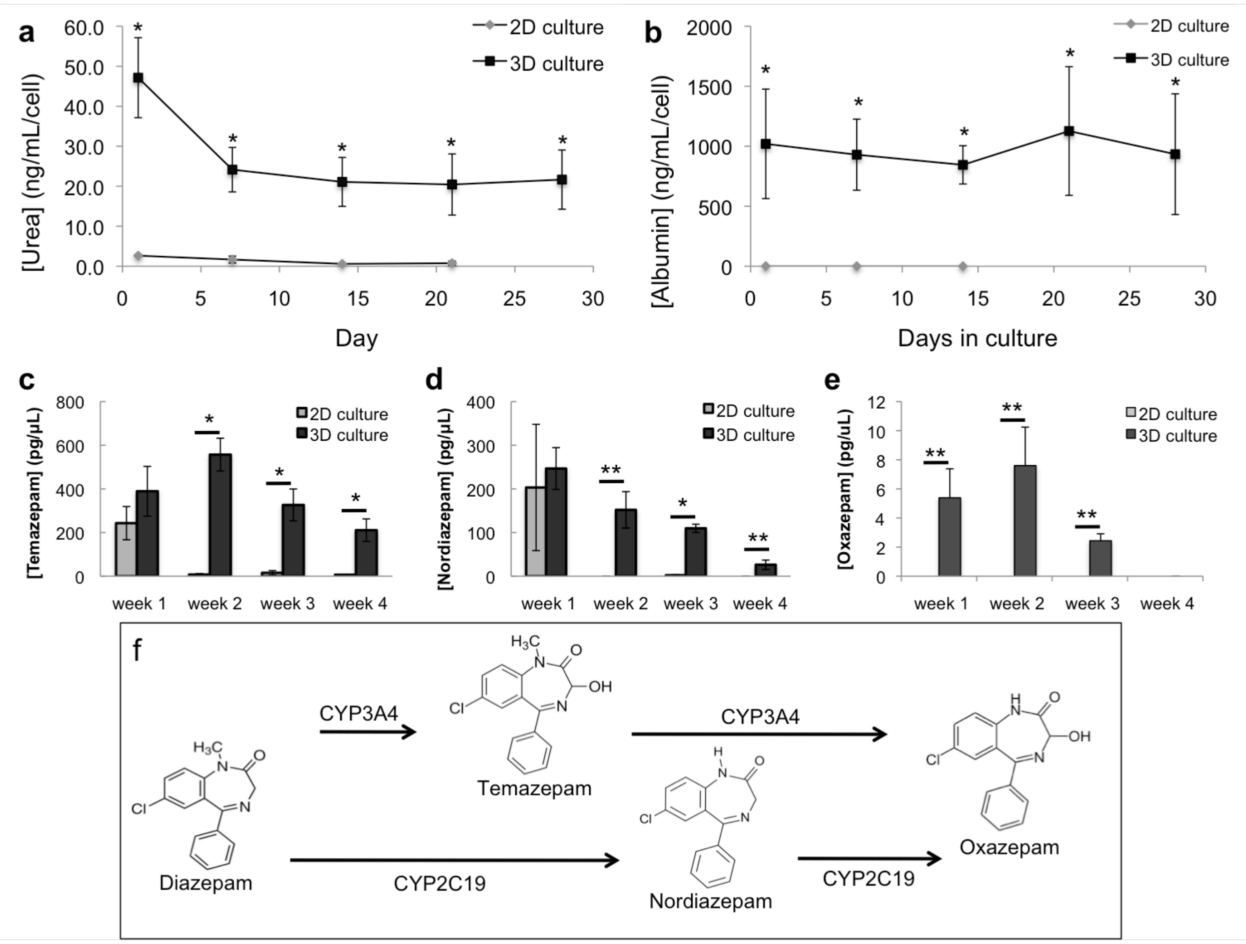

Supplementary Figure 3. Liver organoids retain dramatically increased baseline liver function and metabolism compared to 2-D hepatocyte cultures. a-b) Normalized a) albumin and b) urea secretion into media, analyzed by ELISA and colorimetric assays show dramatically increased functional output in the 3-D organoid format in comparison to 2-D hepatocyte sandwich cultures. Quantification of the diazepam metabolites c) temazepam, d) noridazepam, and e) oxazepam primarily by CYP2C19 and CYP3A4. Statistical significance: * $p < 0.05$ between 3-D and 2-D comparisons at each time point. f) Description of liver metabolism of diazepam into temazepam, nordiazepam, and oxazepan by cytochrome p450 isoforms.

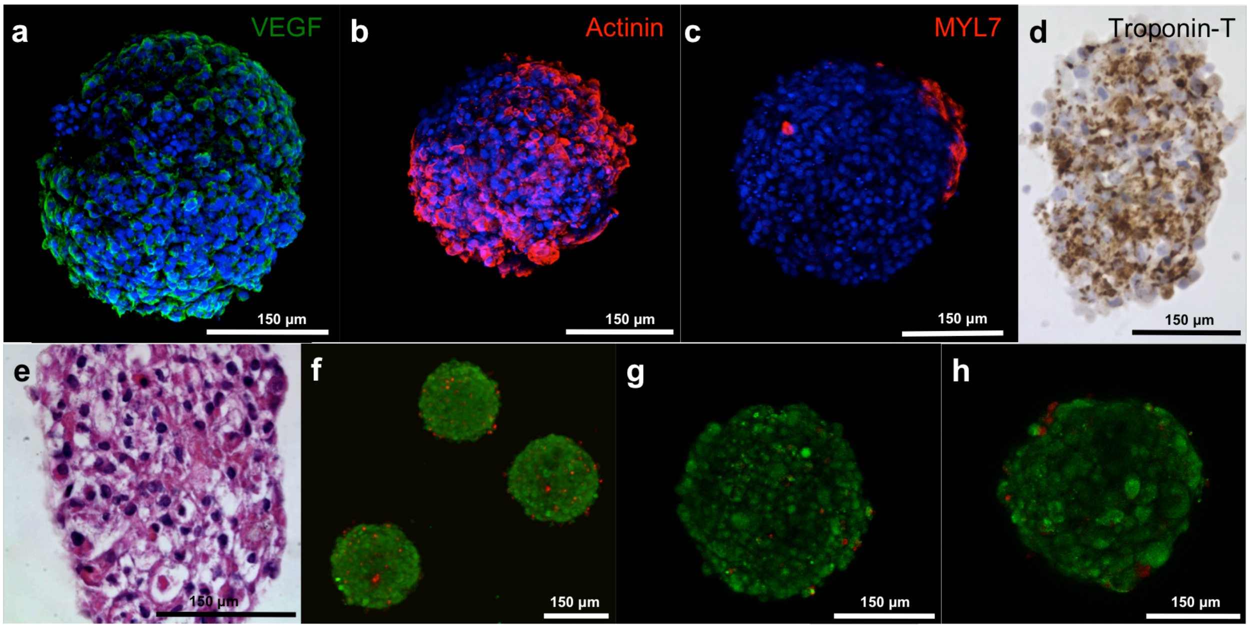

Supplementary Figure 4. Cardiac organoids exhibit cardiac-specific markers and remain stable and viable over time. Cardiac organoids were stained for a) VEGF, b) actinin, c) low levels of myosin regulatory light chain 7 (MYL7) (Red – indicated stain; Blue – DAPI), d) cardiac troponin-T (brown) with hemotoxylin counterstain, e) H&E, and f-h) Live/Dead viability/cytoxocity stains on f) day 1, g) day 28, and h) day 35 of culture. Green – calcein AM-stained viabile cells; Red – ethidium homodimer-stained dead cell nuclei.

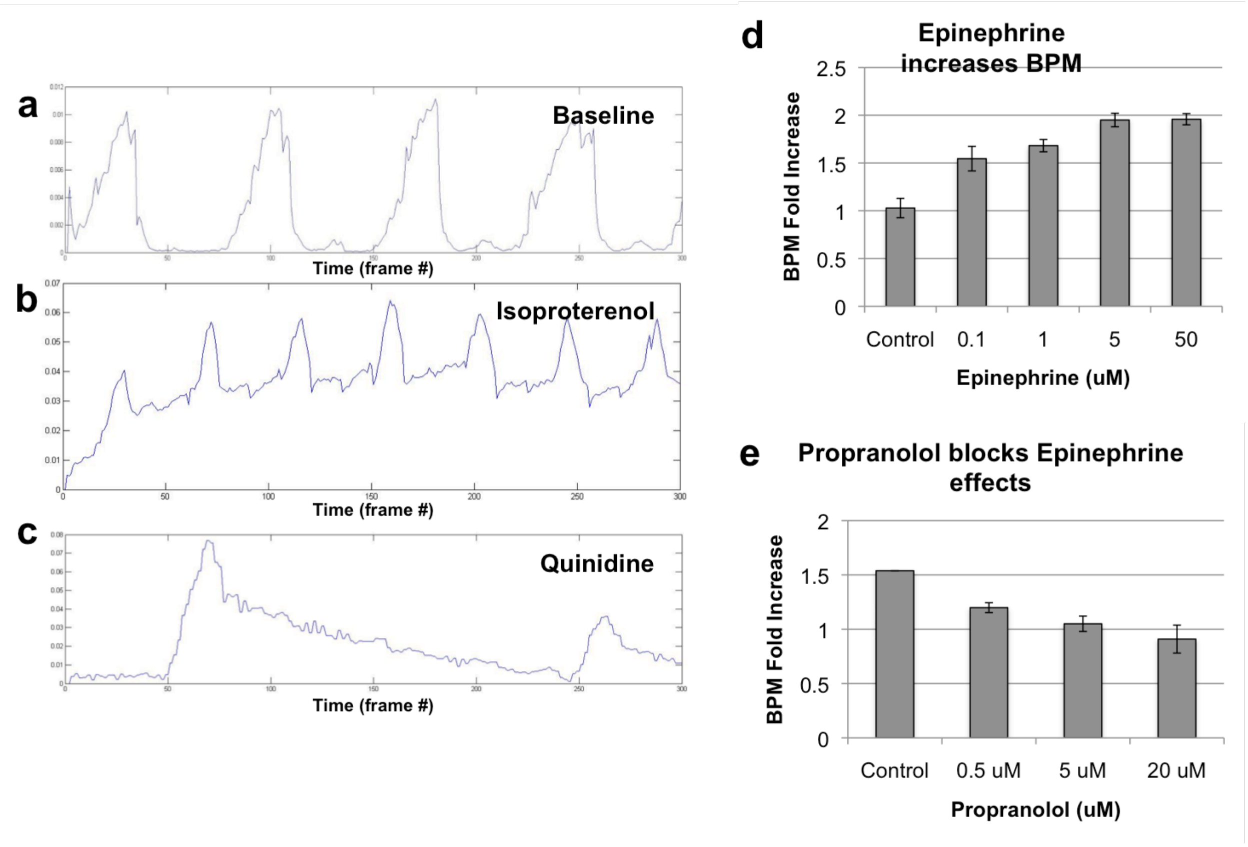

Supplementary Figure 5. Cardiac organoids modulate beat rate in response to drug treatment. a) Beating output under baseline conditions, from which beating rate is determined. Change in cardiac organoid beat rate resulting from exposure to b) isoproterenol, or c) quinidine. d-e) Cardiac organoid response to epinephrine and propranolol. d) Cardiac organoids produce a dose dependent increase in beat rate ranging from 1 to 2-fold with increasing epinephrine concentration before reaching a plateau with 5 μM epinephrine and higher. e) Initial incubation with propranolol concentrations ranging fron 0 to 20 μM results in a dose dependent decrease in beating rate after administration of 5 μM epinephrine.

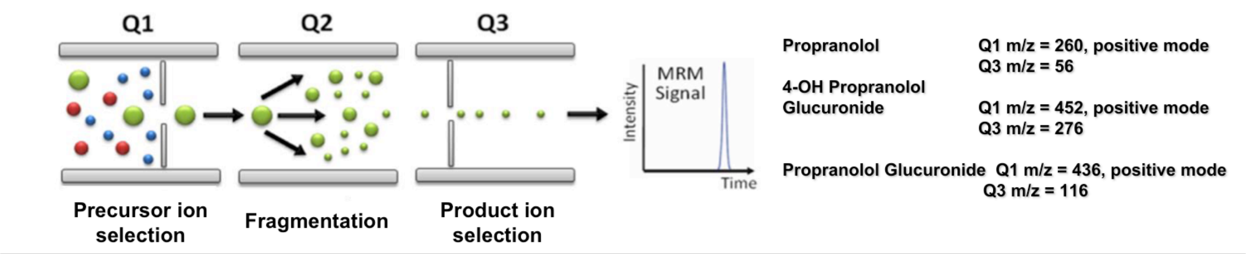

Supplementary Figure 6. Verification of propranolol metabolism by liver organoids. Strategy for targeted detection of propranolol and selected phase I and II metabolites by triple quadrupole LC-MS MRM analysis.

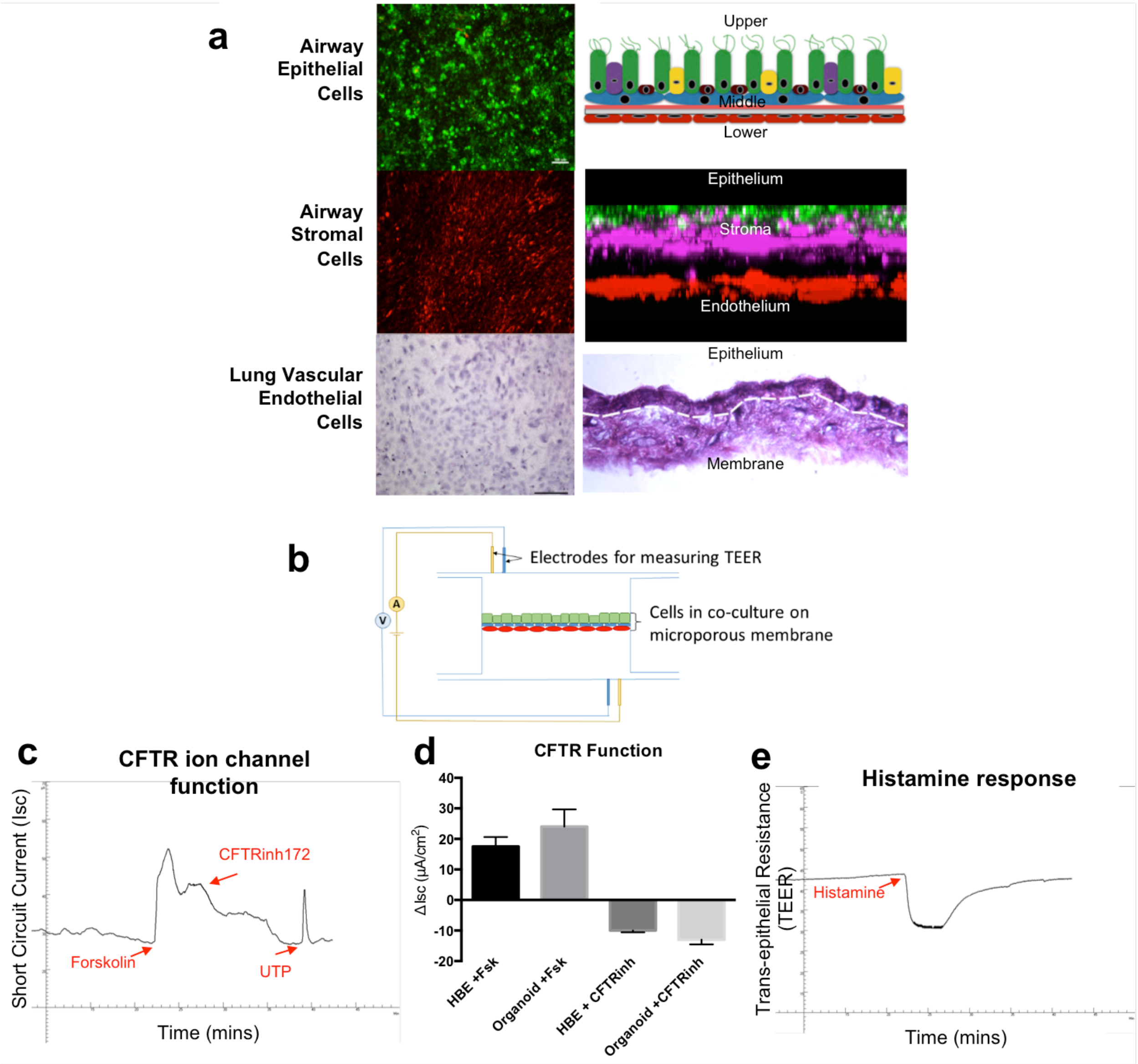

Supplementary Figure 7. Generation of 3D lung organoids for disease modeling, drug development and testing. a) 3D lung organoids are comprised of airway epithelial cells, airway stromal cells, and lung microvascular endothelial cells. The layered 3D organoid rapidly produces a cellular organization similar to that seen in native airway tissue, with a polarized epithelial surface exposed to the air-liquid interface, a stromal component providing tissue structure, and an endothelium forming a thin vascular barrier exposed to liquid media. Layered organoids can be maintained in culture for over 4 weeks with maintenance of transepithelial resistance and cell viability. The advantage of the layered technique of organoid formation is the ability to rapidly establish an organized tissue representing normal airway structure. b) Trans-epithelial electrical resistance monitoring. c) 3D lung organoids demonstrate physiological responses to CFTR activating or inhibitory pharmaceuticals and d) histamine as demonstrated by assessing CFTR ion channel activity by short circuit current (Isc) and TEER.

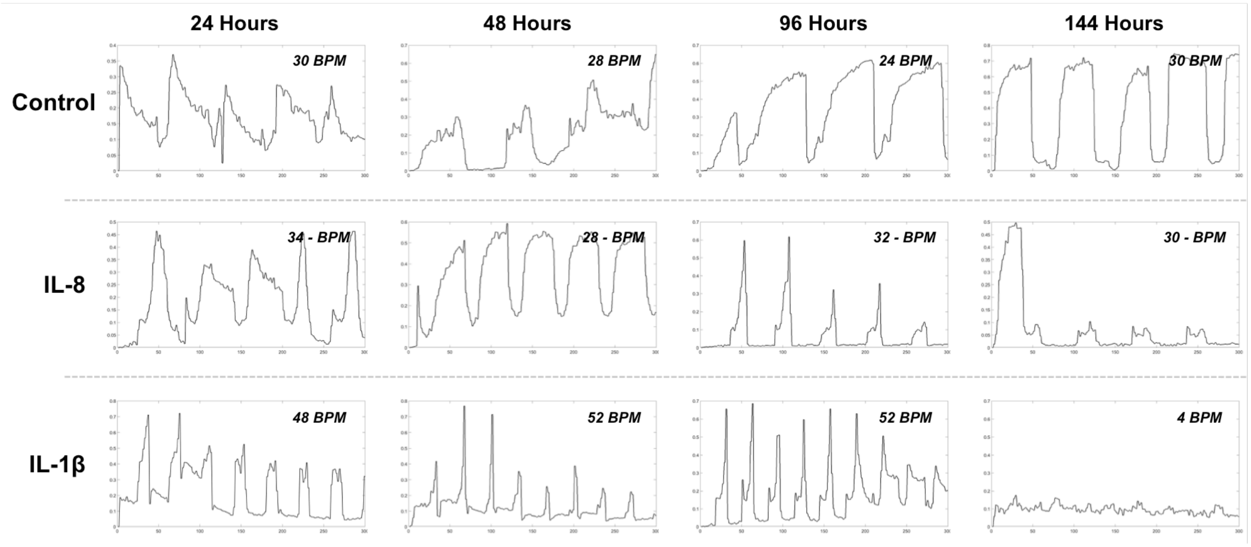

Supplementary Figure 8. Representative cardiac organoid beat plots under baseline conditions, and direct treatment with IL-8 and IL-1β at 24, 48, 96, and 144 hours. Plots shown cover 10 second segments of video files obtained during the course of the experiment.

Supplementary Code Files

Data File S1. MatLab code for pixelation and beat plot output – Executable code.txt

Data File S2. MatLab function file – analyzeDisp.m

Data File S3. MatLab function file – disparityBased2.m

Data File S4. MatLab function file – getAndCrop.m

Data File S5. MatLab function file – getVideoFrames.m

Data File S6. MatLab function file – Video.m

Executable code for MatLab

```
E(:,:,ix) = disparity(ref, I, 'UniquenessThreshold', 30);
E2 = filter2(fspecial('average',5),E(:,:,ix))/255;
E3 = E2 <= 0 | E2 >= 0.55;
E(:,:,ix) = imcomplement(E3);
Emean(1,2*ix - 1) = double(mean(mean(E(:,:,ix))))
Emean(1,2*ix) = double(mean(mean(E(:,:,ix))));
plot(Emean);
```

analyzeDisp.m

```
function [Emean, E] = analyzeDisp(cropeddir)


d=dir(strcat(cropeddir,'\*.jpg'));
totcordt = transpose([1;0;0]);
for ix=1:length(d)

  fn=d(ix).name
  [pathstr,name,ext] = fileparts(fn)
  fnI = strcat(cropeddir,'\' , name , '.jpg');

  rgb = imread(strcat(fnI));
  % figure
  % imshow(rgb)
  gray1 = rgb2gray(rgb);
  % imshow(gray1)
  I = gray1;
  if (ix == 1)
      ref = I;
  end

  E(:,:,ix) = disparity(ref, I, 'UniquenessThreshold', 30);
  E2 = filter2(fspecial('average',5),E(:,:,ix))/255;
  E3 = E2 <= 0 | E2 >= 0.55;
  E(:,:,ix) = imcomplement(E3);
  Emean(1,2*ix - 1) = double(mean(mean(E(:,:,ix))))
  Emean(1,2*ix) = double(mean(mean(E(:,:,ix))));
end

[minval, index] = findpeaks(Emean, 'minpeakdistance', 8, 'minpeakheight',
.7*(max(Emean)));


plot(Emean);
% hold on; plot(index, minval, 'k^', 'markerfacecolor', [1 0 0])
```

disparityBased2.m

```
function [E, Emean] = disparityBased2(vidname)

% Dependent functions: getVideoFrames, getAndCrop, analyzeDisp

maindir = strcat('D:\Downloads\Cardiac\drive-download-20170222T170517Z\Raw
files\',vidname);
```

```
framesdir = strcat('D:\Downloads\Cardiac\drive-download-20170222T170517Z\Raw
files\',vidname,'\frames');
cropeddir = strcat('D:\Downloads\Cardiac\drive-download-20170222T170517Z\Raw
files\',vidname,'\croped');
procdir = strcat('D:\Downloads\Cardiac\drive-download-20170222T170517Z\Raw
files\',vidname,'\proc');

[s,mess,messid] = mkdir(maindir);
[s,mess,messid] = mkdir(framesdir);
[s,mess,messid] = mkdir(cropeddir);
[s,mess,messid] = mkdir(procdir);

getAndCrop(vidname, framesdir, cropeddir);

[Emean, E] = analyzeDisp(cropeddir);
```

getAndCrop.m

```
function getAndCrop(vidname, framesdir, cropeddir)

getVideoFrames(strcat(vidname,'.MP4'),framesdir);
d=dir(strcat(framesdir,'\*.jpg'));
I = imread(strcat(framesdir,'\' , d(1).name));
[I2 rect] = imcrop(I);
imshow(I), figure, imshow(I2)
for ix=1:length(d)

  fn=d(ix).name
  [pathstr,name,ext] = fileparts(fn)
  fnI = strcat(framesdir,'\' , name , '.jpg');
  I = imread(strcat(fnI));
  J = imcrop(I,rect);
  imwrite(J,strcat(cropeddir,'\',name,'.jpg'));

end
```

getVideoFrames.m

```
function imgs = getVideoFrames(vid, savetodir, startt, endt, step)

% extracts frames from a video and saves them as image files in a given folder.
%
% create: 5/9/2014 (earliest known)
% modified: 12/10/2014 [fix a bug for output directory]
% author(s): Dongxi Zheng
```

```
%
% description
%   This function generates a series of images corresponding to specified
%   frames of a given video file. Users can specify the time in second of
%   the starting frame and the time in second of the ending frame as well
%   as the time lap in second between two successive frames. Users can also
%   specify in which folder to store these images.
%
%   inputs:
%   vid - a VideoReader object or a path to a video file
%   from - the starting second of the extracted frames (default 0)
%   to - the ending second of the extracted frames (default end time of video)
%   step - the interval in seconds between frames (default 5)
%   savetodir - (optional) the output directory (default a
%              "tmp-extracted-frames" subfolder of the video file's
%              directory or of the current execution directory)
% outputs:
%   imgs - heigth X width X colors X frames arrays representing the
%         extracted frames.



  % limit input from 0 to 5 arguments
  narginchk(0, 5);

  % constants for default values
  DEFAULTSTARTT = 0; % default start time = 1 seconds
  DEFAULTSTEP = .033; % default step = 5 seconds
  MINSTEP = 10^(-9); % minimum step = 1 nanosecond
  DEFAUTOUTDIRNAME = 'tmp-extracted-frames'; % default output dir name

  % read the video file and its properties
  if nargin < 1
    [fn, fp, ~] = ...
      uigetfile('*','Please choose the video file to be processed.');
    vid = [fp, fn];
  end
  if isa(vid, 'VideoReader')
    vidObj = vid;
  else
    vidObj = VideoReader(vid);
  end

  frameRate = vidObj.FrameRate;
  duration = vidObj.Duration;
```

```
% nFrames = vidObj.NumberOfFrames;
[nrow, ncol, nclr] = size(read(vidObj, 1));

% vidHeight = xyloObj.Height;
% vidWidth = xyloObj.Width;

% set default values to the rest arguments when no specified
% set default start frame time
if nargin < 3
   startt = DEFAULTSTARTT;
   userinput = input(['Type in the time in seconds of the starting ', ...
                      'frame, \nor simply enter to accept ', ...
                      'default (', num2str(startt), ' seconds): '],'s');
   tmpstartt = str2double(userinput);
   if (~isnan(tmpstartt))
      startt = min(max(0,tmpstartt),duration);
   end
end

% set default end frame time
if nargin < 4
   endt = duration;
   userinput = input(['Type in the time in seconds of the stopping', ...
                      'frame, \nor simply enter to accept ', ...
                      'default (', num2str(endt), ' seconds): '],'s');
   tmpendt = str2double(userinput);
   if (~isnan(tmpendt))
      endt = min(max(startt,tmpendt),duration);
   end
end

% set default frame interval (step)
if nargin < 5
   step = DEFAULTSTEP;
   userinput = input(['Type in the time in seconds between two ', ...
                      'extracted frames, \nor simply enter to accept ', ...
                      'default (', num2str(step), ' seconds): '],'s');
   tmpstep = str2double(userinput);
   if (~isnan(tmpstep))
      step = min(max(MINSTEP,tmpstep),endt-startt);
   end
end

% set default savetodir value
if nargin < 2
   videopath = './';
```

```
    if (isa(vid, 'char')
        [videopath,~,~] = fileparts(vid);
    end
    savetodir = fullfile(videopath,DEFAUTOUTDIRNAME);
    userinput = input(['Do you want to set the output directory?\n', ...
                       '(The default is: "', strrep(savetodir,'\','/'), ...
                       '")\n', '(y/n): '],'s');
    if strcmp(userinput, 'y')
        savetodir = uigetdir;
    end
end

% calculate the start and end frame indices and the number of frames to
% skip each time
startF = round(frameRate*startt)+1;
endF = round(frameRate*endt);
stepF = round(frameRate*step)+1;
disp(['Extracting frames from ',vidObj.Name]);
disp(['starting at frame no.', num2str(startF), ...
      ' (at second ', num2str(startt), '),']);
disp(['stopping at frame no.', num2str(endF), ...
      ' (at second ', num2str(endt), '),']);
disp(['and by every other ', num2str(stepF), ' frames (', ...
      num2str(step), ' seconds).']);
disp(['Output folder: "', savetodir, '"']);

% allocate the memory for storing the frames
imgs = uint8(zeros(nrow, ncol, nclr, floor((endF-startF)/stepF)+1));

% read one frame at a time.
count = 0;
for k = startF:stepF:endF
    count = count+1;
    imgs(:,:,:,count) = read(vidObj, k);
end

% write images to files
if exist('savetodir','var')
    if (~exist(savetodir,'dir'))
        mkdir(savetodir);
    end
    count = 0;
    padding = power(10,size(num2str(max(startF, endF)),2));
    for k = startF:stepF:endF
        count = count+1;
        padded_k = num2str(padding+k);
```

```
            img = imgs(:,:,:,count);
            imwrite(img, [savetodir,'\frame',padded_k(2:end),'.jpg'], 'JPG');
        end
    end
end
```

Video.m

```
function [Evid] = Video(E, fps)

[x , y, t] = size(E);

for i=1:t
    axis tight manual;
       imshow(E(:,:,i))
       Evid(i)=getframe;
end

movie2avi(Evid, 'Ricin 100nM', 'fps', fps, 'compression', 'none')
```

Supplemental Movie Files

Movie S1. Cardiac – baseline condition video

Movie S2. Cardiac – baseline condition black and white moving pixels quantification video

Movie S3. Cardiac – epinephrine video

Movie S4. Cardiac – propranolol and epinephrine video

Movie S5. Cardiac – metabolized propranolol and epinephrine video
